# Non-conventional graphene superlattices as electron band-pass filters

**DOI:** 10.1038/s41598-019-45417-3

**Published:** 2019-06-19

**Authors:** A. Sánchez-Arellano, J. Madrigal-Melchor, I. Rodríguez-Vargas

**Affiliations:** 1 Unidad Académica de Física, Universidad Autónoma de Zacatecas, Calzada Solidaridad Esquina Con Paseo La Bufa S/N, 98060 Zacatecas, Zac. Mexico; 2 Unidad Académica de Ciencia y Tecnología de la Luz y la Materia, Universidad Autónoma de Zacatecas, Carretera Zacatecas-Guadalajara Km. 6, Ejido La Escondida, 98160 Zacatecas, Zac. Mexico

**Keywords:** Electronic properties and materials, Electronic and spintronic devices

## Abstract

Electron transmission through different non-conventional (non-uniform barrier height) gated and gapped graphene superlattices (GSLs) is studied. Linear, Gaussian, Lorentzian and Pöschl-Teller superlattice potential profiles have been assessed. A relativistic description of electrons in graphene as well as the transfer matrix method have been used to obtain the transmission properties. We find that it is not possible to have perfect or nearly perfect pass bands in gated GSLs. Regardless of the potential profile and the number of barriers there are remanent oscillations in the transmission bands. On the contrary, nearly perfect pass bands are obtained for gapped GSLs. The Gaussian profile is the best option when the number of barriers is reduced, and there is practically no difference among the profiles for large number of barriers. We also find that both gated and gapped GSLs can work as omnidirectional band-pass filters. In the case of gated Gaussian GSLs the omnidirectional range goes from −50° to 50° with an energy bandwidth of 55 meV, while for gapped Gaussian GSLs the range goes from −80° to 80° with a bandwidth of 40 meV. Here, it is important that the energy range does not include remanent oscillations. On the light of these results, the hole states inside the barriers of gated GSLs are not beneficial for band-pass filtering. So, the flatness of the pass bands is determined by the superlattice potential profile and the chiral nature of the charge carriers in graphene. Moreover, the width and the number of electron pass bands can be modulated through the superlattice structural parameters. We consider that our findings can be useful to design electron filters based on non-conventional GSLs.

## Introduction

Semiconductor superlattices are essential as injector and/or active region in several applications. In quantum cascade lasers^1,2^, superlattices with well-defined stop (high reflection) and pass (high transmission) bands are needed for good device efficiency. Since its seminal proposal^3,4^, semiconductor superlattices with Gaussian potential profile are regarded as the archetypal structure to obtain flat stop and pass bands with nearly 100% reflection and transmission probability. The fundamental properties of these non-uniform barrier height (non-conventional) superlattices were experimentally verified^5^. The electron-electron interaction and disorder effects were also studied^5,6^, having a limited influence on the filtering characteristics. The striking features of the so-called Gaussian superlattices sparked a lot of interest, deriving in multiple studies^5–15^. These studies have been extended to other elemental excitations^16–21^. Recently, non-conventional potential profiles have been used to improve the efficiency of thermoelectric devices and magnetic tunnelling junctions^22–24^. The common factor in all these studies is the use of the Gaussian profile or alike profiles to create nearly perfect stop and pass bands.

Under this context, a possible electronics based on cutting-edge materials like graphene will need efficient devices that act as injector, collector and/or active region. In fact, the so-called gated GSLs^25–27^, a graphene sheet with a periodic arrangement of top gates, represent a possibility. In these superlattices the propagation of charge carriers is highly anisotropic^25,26^. The energy minibands and minigaps depend strongly on the angle of the impinging electrons, opening the possibility for an angle-dependent bandgap engineering^27^. The transmission minigaps (stop bands) can be tuned from meV to eV by changing the angle of incidence. An alternative is to fix the angle of incidence and enlarge the stop bands by using two or more gated GSLs with different periodic potentials^28^. To get the stop-band enlargement it is fundamental that the bandgaps of the constituent superlattices overlap. Another possibility are aperiodic and non-conventional gapped graphene heterostructures^29–31^. In this case, the omnidirectional electronic bandgap associated to gapped graphene can be extended by appropriately choosing the widths of the potentials of the constituent superlattices^29,30^. It is also possible to have a tunable band-stop filter by modulating the Fermi velocity barriers and the external bias voltage in non-conventional fashion^31^.

As we have corroborated much attention has been paid to create and modulate stop bands in graphene superlattices. However, for superlattice devices pass bands are as important as stop bands. Unfortunately, the literature about pass bands in GSLs, specifically about how flattening them^32^, is scarce. For instance, magnetic GSLs with Gaussian modulation in the heights of the magnetic barriers could be an option for band-pass filters^32^. However, magnetic modulation with precise potential profiles could be tricky. Actually, gating is a more natural technique for 2D materials. In fact, most of the exotic and unprecedented phenomena in graphene have been tested with the help of this technique. With gating we will have electrostatic barriers with hole states inside them. These states are fundamental for most of the exotic phenomena in graphene, like Klein tunnelling^33,34^. However, as far as we know, the role of them in band-pass filtering has not been addressed. Other issue that has been controversial since the beginning of non-conventional superlattices is the shape of the potential profile^3,7,12,30^. Some works claim that Gaussian superlattices are the best for band-pass filtering^3^, others say that there is nothing special with the Gaussian profile and that any potential with smooth variation can serve as good band-pass filter^7,30^.

The aim of the present work is twofold: first, we want to know the relevance of hole states for band-pass filtering, and in order to do so, we will compare the transmission properties of two antagonistic systems, gated and gapped non-conventional GSLs; second, we want to find out to what extent the shape of the potential profile is preponderant for the filtering characteristics, so we will consider different non-conventional GSLs such as Gaussian, Lorentzian, Linear and Pöschl-Teller.

## Model and Method

**Figure 1:**
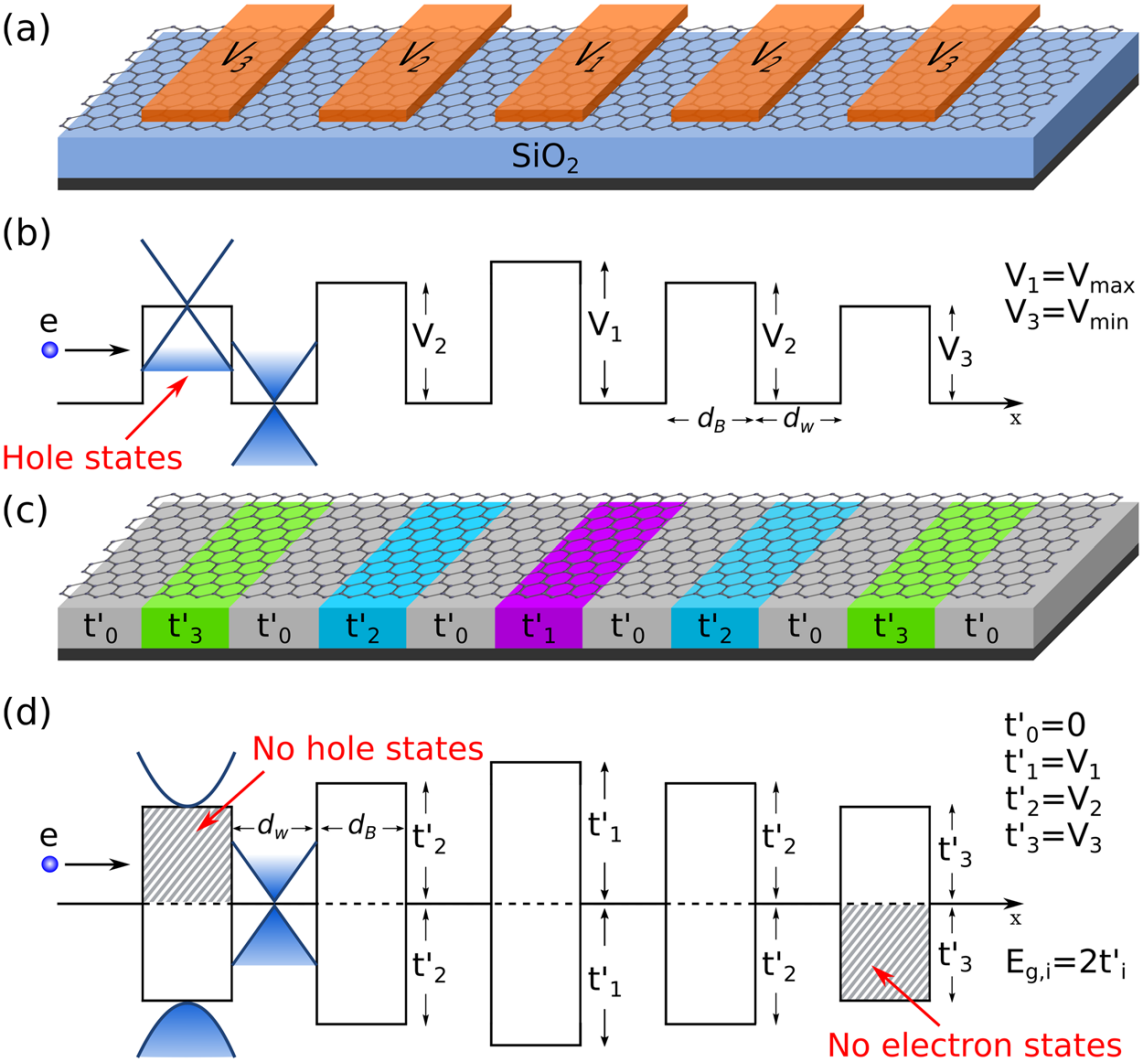
(**a**) Schematic representation (top view) of the possible gated GSLs. The orange stripes represent metallic electrodes at different potential energies. (**b**) Resulting potential profile of (**a**). Here, *V*_1_ and *V*_3_ represent the maximum (*V*_*max*_) and minimum (*V*_*min*_) potential barriers in the system, and *d*_*B*_ and *d*_*W*_ the widths of the barrier and well regions. (**c**) and (**d**) are the same as in (**a**) and (**b**), but for gapped GSLs. In this case the potential barriers are generated by substrates with different degree of interaction with the graphene sheet (${t^{\prime} }_{i}$). The main difference between gated and gapped GSLs is that in the former there are hole states inside the barriers, while in the latter the bandgaps $E{g}_{i}=2{t^{\prime} }_{i}$ prohibit the existence of hole states (*E* > 0) and electron states (*E* < 0) inside the barriers. By appropriately choosing the heights of the potential barriers we can obtain superlattices with Linear, Gaussian, Lorentzian and Pöschl-Teller potential profiles, see Fig. 2.

**Figure 2:**
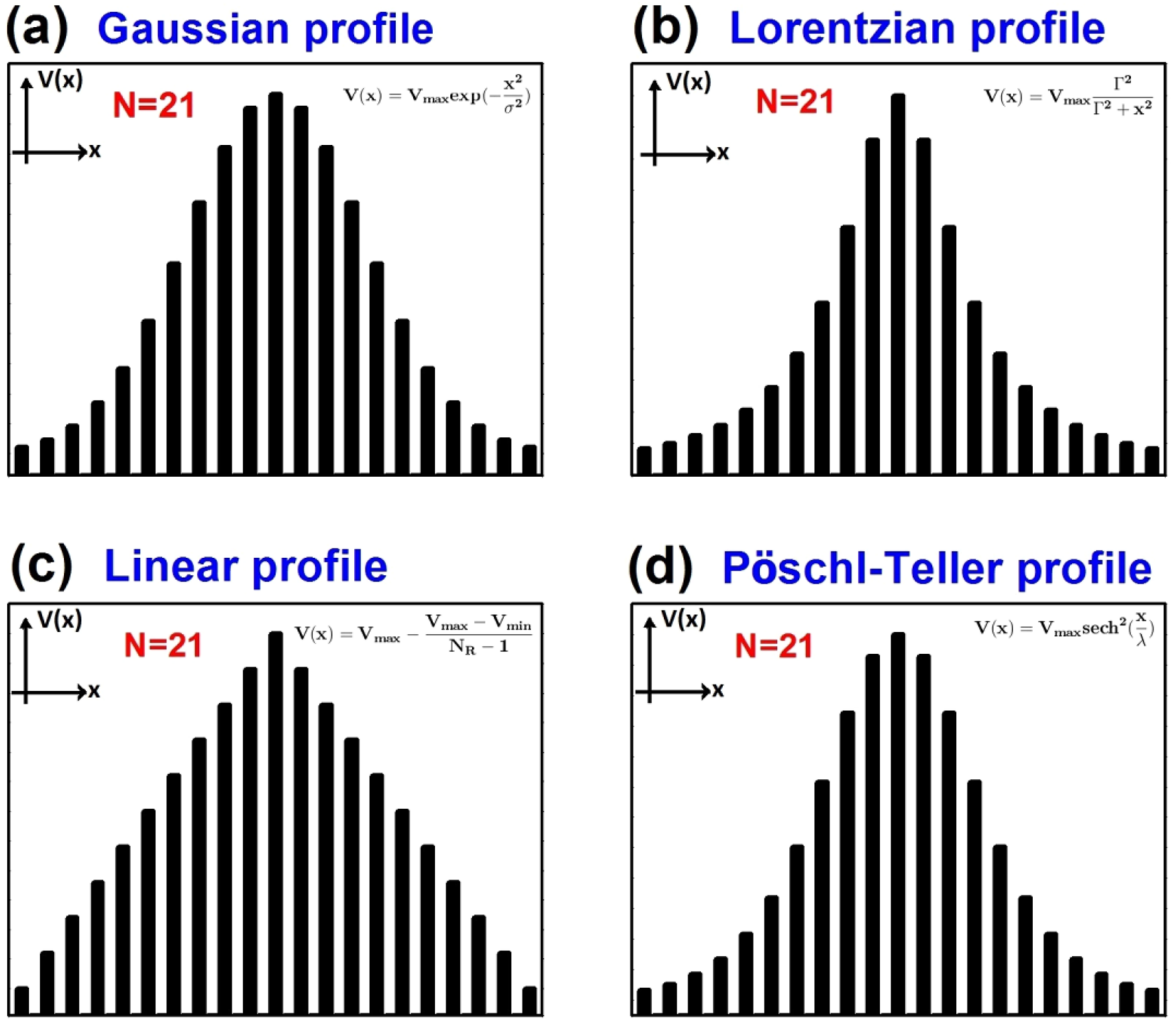
(**a**) Gaussian, (**b**) Lorentzian, (**c**) Linear and (**d**) Pöschl-Teller potential profiles of gated and gapped GSLs. In all cases the number of barriers is *N* = 21. The height of the potential varies in Gaussian, Lorentzian, Linear and Pöschl-Teller fashion going from a minimum value at the edges of the structure to a maximum value at the center of it. The mathematical function used in each case is also reported. Here, the function parameters (*σ*, Γ, *N*_*R*_ and *λ*) are adjusted to the total length of the superlattice so that the minimum and maximum barrier heights is the same for all profiles.

The schematic representation of the possible devices for non-conventional gated and gapped graphene superlattices is shown in Fig. 1. In the case of gated GSLs (Fig. 1a) the graphene sheet is placed on a non-interacting substrate like SiO_2_ and top gates are patterned to induce the non-conventional potential profile (Fig. 1b). The device also includes, not shown, a back gate and left and right leads. We can have potential barriers of different height by modulating the strength of the applied electrostatic field. It is also important to remark that there are hole states inside the barriers because the main effect of gating is a shifting of the Dirac cones, see Fig. 1b. In the case of gapped GSLs (Fig. 1c) the potential barriers are induced by substrates with different degree of interaction with the graphene sheet. A possibility to generate the potential barriers could be the so-called heterostructured SiO_2_/SiC substrates^35^. In the SiO_2_ regions the Dirac cone structure of pristine graphene is preserved, while in the SiC regions the interaction between the graphene sheet and the substrate results in a bandgap opening^36^. The breaking of the sublattice symmetry of graphene is the main reason of the bandgap opening^36,37^. The different bandgap sizes necessary to get the non-conventional profiles could be achieved by playing with the termination of the substrate faces, the stoichiometry of SiC and/or the number of graphene layers^36^. Here, it is important to remark that half of the size of the bandgap results in barriers for electrons, and the other half in barriers for holes. Hence, we have the same potential profile for electrons (*E* > 0, with no hole states inside the barriers) and holes (*E* < 0, with no electron states inside the barriers), see Fig. 1d. So, by gating or interacting substrates, in principle, is possible to have non-conventional gated and gapped GSLs, respectively. In our case, we will consider Gaussian, Lorentzian, Linear and Pöschl-Teller potential profiles, see Fig. 2.

The semi-infinite and quantum well regions, in both gated and gapped GSLs, are described by the well-known monolayer graphene Hamiltonian ${H}_{free}=\hslash {v}_{F}\sigma \cdot k$^38,39^, with linear dispersion relation $E=\pm \hslash {v}_{F}k$ and eigenfunctions,

1 $${\psi }_{k}^{\pm }(x,y)=\frac{1}{\sqrt{2}}(\begin{array}{c}1\\ {u}_{\pm }\end{array}){e}^{\pm i{k}_{x}x+i{k}_{y}y},$$

where *v*_*F*_ is the Fermi velocity, *k* is the magnitude of the wave vector **k** in these regions, *k*_*x*_ and *k*_*y*_ are the longitudinal and transverse components of *k*, and *u*_±_ = ±sgn(*E*)*e*^±*iθ*^ are the coefficients of the wave functions that depend on the angle of the impinging electrons, *θ* = arctan(*k*_*y*_/*k*_*x*_). The Hamiltonian for the gated regions is ${H}_{gated}=\hslash {v}_{F}\sigma \cdot {q}_{i}+{V}_{i}1$, with dispersion relation $E-{V}_{i}=\pm \hslash {v}_{F}{q}_{i}$ and eigenfunctions,

2 $${\psi }_{{q}_{i}}^{\pm }(x,y)=\frac{1}{\sqrt{2}}(\begin{array}{c}1\\ {v}_{\pm ,i}\end{array}){e}^{\pm i{q}_{x,i}x+i{q}_{y,i}y},$$

here *V*_*i*_ represents the strength of the electrostatic potential of the *i*-th barrier, *q*_*i*_ is the magnitude of the corresponding wave vector **q**_***i***_, *q*_*x*,*i*_ and *q*_*y*,*i*_ are the components of *q*_*i*_, and *v*_±,*i*_ the coefficients of the wave functions^35^. For the gapped regions ${H}_{gapped}=\hslash {v}_{F}\sigma \cdot {q}_{i}+{t^{\prime} }_{i}{\sigma }_{z}$ and ${E}^{2}-{t^{\prime} }_{i}^{2}={\hslash }^{2}{v}_{F}^{2}{q}_{i}^{2}$. The eigenfunctions have the same mathematical form as Eq. (2), however *q*_*x*,*i*_ and *v*_±,*i*_ depend on the bandgap energy ${t^{\prime} }_{i}={E}_{g,i}/2$^35^.

This information, eigenfunctions and wave vectors, is essential to compute the transfer matrix of the system, and with it the transmission probability or transmittance. By imposing the continuity condition for the wave function along the superlattice structure as well as the conservation of the transverse momentum, we can obtain the transfer matrix of the system. For a non-conventional GSL of five barriers (*N* = 5) the transfer matrix can be written as

3 $$M={M}_{3}{M}_{W}{M}_{2}{M}_{W}{M}_{1}{M}_{W}{M}_{2}{M}_{W}{M}_{3},$$

where *M*_*W*_ and *M*_*i*_ are the transfer matrices of the well and barrier (*i*-th) regions. As our structure is symmetric with respect to the central barrier there are only three distinct transfer matrices of the barriers *M*_1_, *M*_2_ and *M*_3_ in the case of *N* = 5. The transfer matrices of wells and barriers depend on the so-called dynamic and propagation matrices^35^. The energy and angle dependent transmittance can be obtained through the (1, 1) element of the transfer matrix

4 $$T(E,\theta )=\frac{1}{|{M}_{11}{|}^{2}}.$$

## Results and Discussion

**Figure 3:**
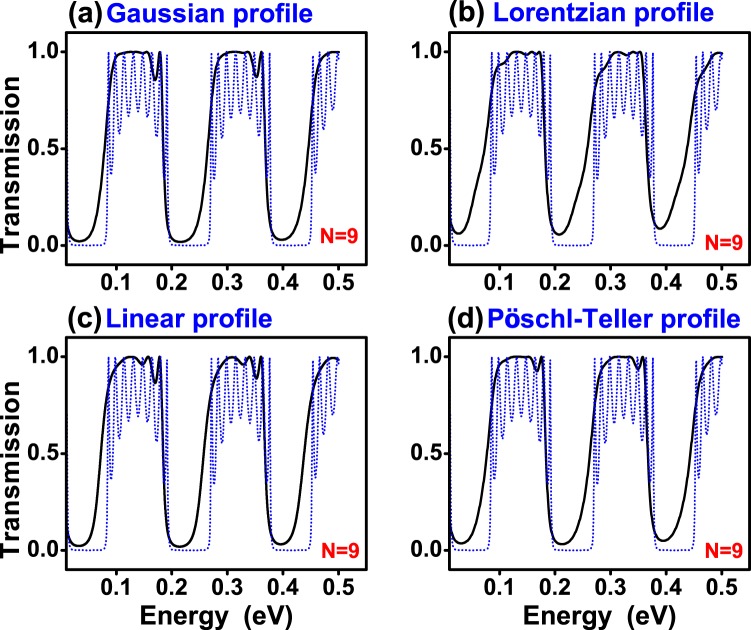
Transmission probability or transmittance versus the energy of the incident electrons for gated graphene superlattices with (**a**) Gaussian, (**b**) Lorentzian, (**c**) Linear and (**d**) Pöschl-Teller potential profiles. In all cases the number of barriers, the heights of the maximum and minimum potential barriers, the widths of the barrier and well regions as well as the angle of incidence were the same. The specific values for all these quantities were *N* = 9, *V*_*max*_ = 0.13 eV, *V*_*min*_ = 0.01 eV, *d*_*B*_ = 20*a*, *d*_*W*_ = 80*a* and *θ* = 45°, respectively. Here, *a* represents the carbon-carbon distance in graphene, 0.142 nm.The dotted-blue curves correspond to the transmittance of uniform gated GSLs. The height of the uniform barriers is *V*_*max*_. The other structural parameters are the same as for non-conventional GSLs.

**Figure 4:**
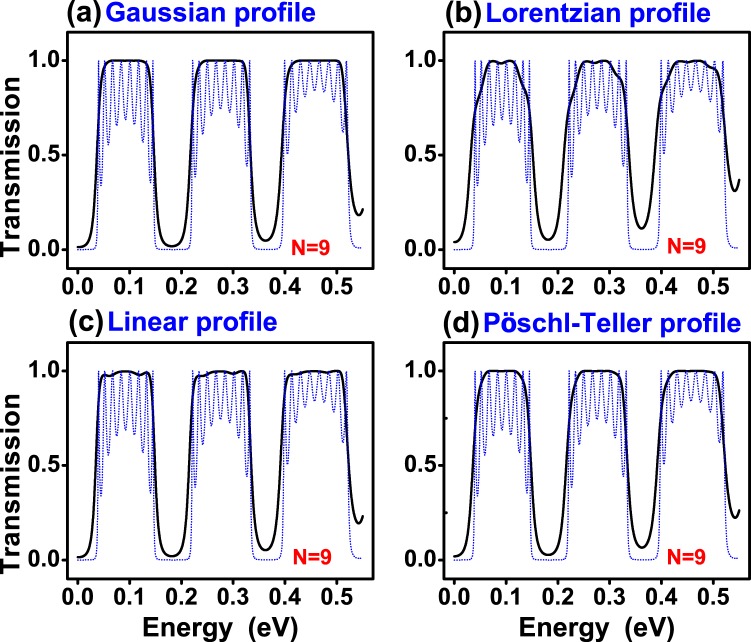
The same as in Fig. 3, but for gapped GSLs. Here, all ${t^{\prime} }_{i}$ are the same as the *V*_*i*_ of gated GSLs. In particular, ${t^{\prime} }_{max}={V}_{max}=0.13$ eV and ${t^{\prime} }_{min}={V}_{min}=0.01$ eV. The dotted-blue lines represent the transmittance of uniform gapped GSLs.

**Figure 5:**
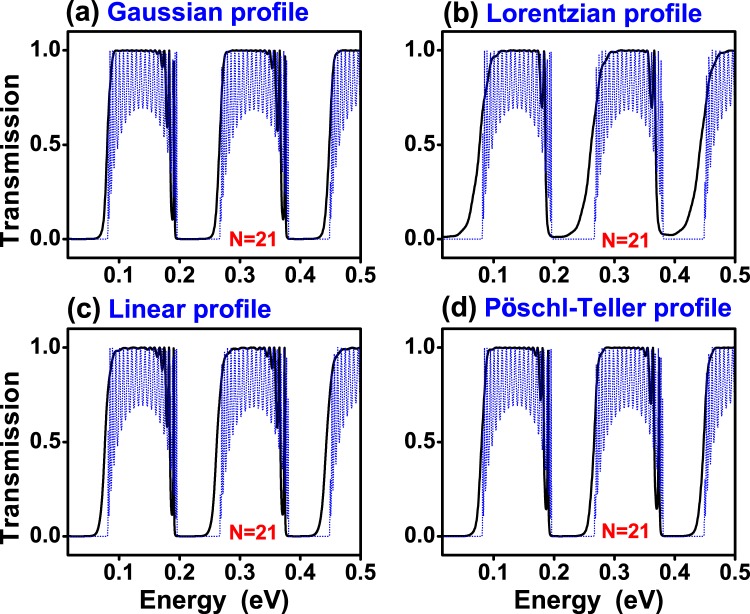
The same as in Fig. 3, but for *N* = 21.

**Figure 6:**
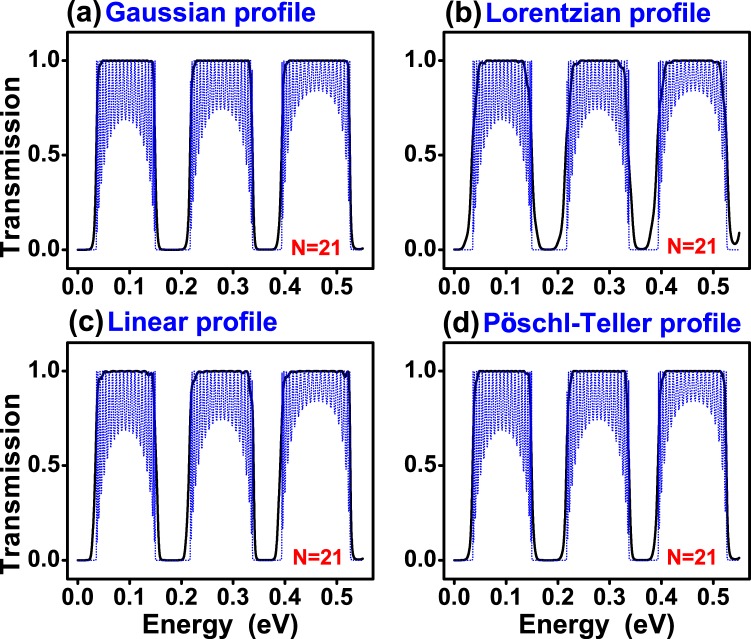
The same as in Fig. 4, but for *N* = 21.

In Fig. 3 we show the transmittance of (a) Gaussian, (b) Lorentzian, (c) Linear and (d) Pöschl-Teller gated GSLs. We consider the same structural parameters for all non-conventional gated GSLs. In concrete, the number of barriers, the maximum and minimum height of barriers, the width of barriers and wells as well as the angle of incidence considered were: *N* = 9, *V*_*max*_ = 0.13 eV, *V*_*min*_ = 0.01 eV, *d*_*B*_ = 20*a*, *d*_*W*_ = 80*a* and *θ* = 45°, respectively. The widths are given in terms of the carbon-carbon distance in graphene *a* = 0.142 nm. We have included the transmittance of a uniform gated GSL, dotted-blue curves, as reference. As we can notice practically all non-conventional profiles reduce notably the transmittance oscillations of the uniform superlattice. We can also see that, regardless of the potential profile, the pass bands present a high probability, close to 100%. However, the pass bands are not well-defined, they have a rounded shape. It is also notice that the quality of the stop and pass bands diminish as the energy increases. Actually, that is a general characteristic of finite superlattices. In fact, as the energy increases the propagation of electrons is less influenced by the superlattice potential. A possible strategy to improve the quality of stop and pass bands in a specific energy range is to increase the number of barriers, see the results for N = 21. For Gaussian and Pöschl-Teller GSLs a notch (small oscillation) arises at the high energy side of the pass bands. Linear GSLs have more than a notch (more oscillations) and Lorentzian GSLs have no oscillations, however the pass bands and stop bands are not as defined as in the other cases. In the case of gapped GSLs (Fig. 4) the pass bands have practically no oscillations and are better defined for the Gaussian profile. Here, it is important to mention that the parameters of gapped GSLs are the same as the ones of gated GSLs. In particular, the *t*′_*i*_’s are the same as the *V*_*i*_’s, specifically ${t^{\prime} }_{max}={V}_{max}=0.13$ eV and ${t^{\prime} }_{min}={V}_{min}=0.01$ eV. In Fig. 5 we show the transmittance of gated GSLs for *N* = 21. As we can see, increasing the number of barriers the oscillations increase and become more pronounced as well as the stop and pass bands adopt a better rectangular form. The Lorentzian profile (Fig. 5b) deserves a special mention because, despite the stop and pass bands not being well-defined as in the other non-conventional GSLs, the remanent oscillations in the pass bands are substantially reduced, in both, number and intensity. In Fig. 6 we show the corresponding results for gapped GSLs. As we can notice the transmission characteristics are nearly the same for all non-conventional gapped GSLs. It is remarkable that the pass bands are essentially flat and with 100% transmission probability. It is also important to highlight the perfect rectangular shape of the stop and pass bands of the Gaussian profile. If we further increase the number of barriers, results not shown, the remanent oscillations of gated GSLs increase even more, and the transmission characteristics, flat pass bands, among non-conventional gapped GSLs become equivalent.

**Figure 7:**
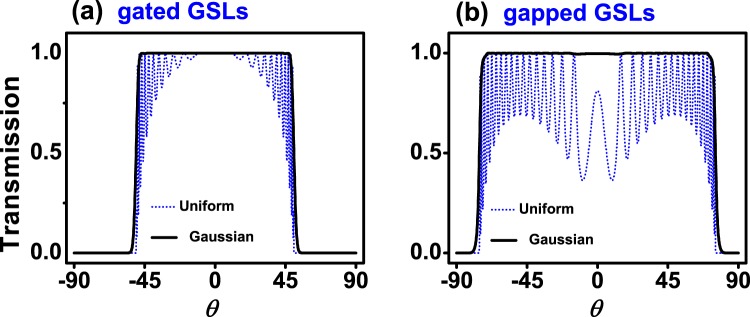
Angular distribution of the transmittance for (**a**) gated and (**b**) gapped Gaussian GSLs. The energy of the impinging electrons is *E* = 0.1 eV. The transmittance for uniform GSLs (dotted-blue curves) has been included as reference. The superlattice parameters are the same as in Figs 5 and 6.

**Figure 8:**
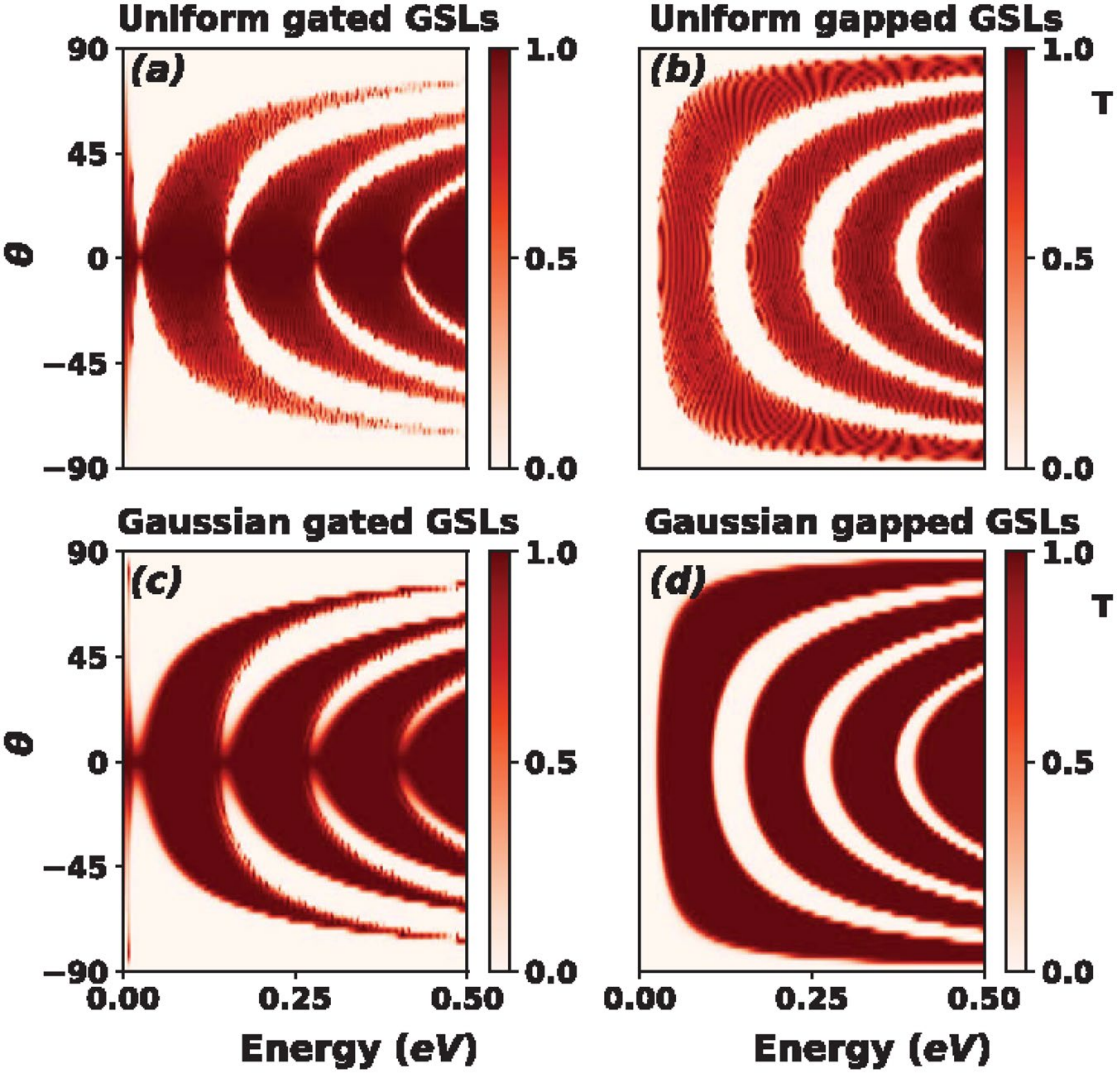
Contour maps (*E*, *θ*) of the transmittance for Uniform ((**a**) gated and (**b**) gapped) and Gaussian ((**c**) gated and (**d**) gapped) GSLs. The superlattice parameters are the same as in Fig. 7.

**Figure 9:**
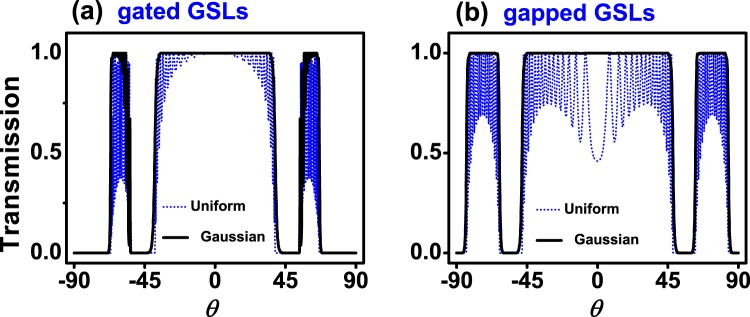
The same as in Fig. 7, but for *E* = 0.23 eV.

If we now fix the energy and vary the angle of incidence we can obtain the so-called angular distribution of the transmittance *T*(*θ*). Figure 7 shows *T*(*θ*) for (a) gated and (b) gapped Gaussian GSLs. We have included the corresponding results for uniform GSLs as reference. The energy of the impinging electrons was fixed to *E* = 0.1 eV, which is lower than the maximum barrier height of the system. The superlattice parameters are the same as in Figs 5 and 6. As in the case of *T*(*E*) the non-conventional profile eliminates the transmittance oscillations present in uniform GSLs. In the gated case we can see a flat pass band that spans from −50° to 50° with no oscillations at the edges. For gapped GSLs the range of perfect transmission is bigger, from −80° to 80°. These results are quite interesting because both gated and gapped non-conventional GSLs can work as omnidirectional filters. Here, it is fundamental to have control of the energy of the impinging electrons. In order to know what happens for other energies as well as to have a bigger picture of the transmission properties we have computed the contour maps (*E*, *θ*) of the transmittance. The corresponding results are shown in Fig. 8. As we can notice the non-conventional profile gives rise to dispersionless transmission bands, uniform dark-red zones. We can also see that the omnidirectional pass bands of Fig. 7 have a considerable energy bandwidth. In particular, the bandwidth for gated GSLs is about 55 meV, while for gapped GSLs is about 40 meV. It is also important to mention that the high energy edge of the pass bands of non-conventional gated GSLs is not as uniform as the low energy edge. This non-uniformity is related to the remanent oscillations previously discussed. This point is quite relevant for gated GSLs because if we want to avoid the remanent oscillations we have to consider energies that do not include the high energy edge of the transmission bands. For instance, if we choose an energy of 0.23 meV, which is above the maximum barrier height, the outermost transmission bands of gated GSLs (Fig. 9a) present remanent oscillations. On the contrary, gapped GSLs (Fig. 9b) present perfect pass bands.

For completeness of the present study we consider that some important remarks are necessary.

Firstly, we can attribute the remanent oscillations to the hole states inside the barriers of gated GSLs because this is the fundamental difference between gated and gapped GSLs. So, further analysis is needed in order to unveil why the oscillations are located in the high energy edge of the transmission bands as well as to design strategies to eliminate them.

Secondly, as in the case of superlattices of conventional materials the transmission characteristics of GSLs can be modulated through the superlattice parameters. For instance, by modulating *d*_*B*_, *d*_*W*_, *V*_*max*_ and *V*_*min*_ we can change the location, width and number of pass bands. However, it is quite important to maintain a good *V*_*max*_/*V*_*min*_ contrast, otherwise we can approximate the structure to a uniform GSL, and consequently the filtering characteristics will be far from a good band-pass filter. In GSLs another parameter that can change the location, number and effective width of pass bands is the angle of incidence. Even more interesting, the Gaussian profile for gapped GSLs is the best for band-pass filtering when the width/spacing of the barriers is adjusted to cover the same total length of a specific (particular *N*) GSL. We have computed different cases for width/spacing of the barriers for gapped GSLs with *N* = 21, and compare them with gated GSLs. The results shown that the Gaussian profile is still the best for band-pass filtering as well as how important is the role of hole states inside the barriers for preserving or not the flatness of the transmission bands. However, it is important to mention that in order to have exceptional flat pass bands it is a matter of a delicate balance between the structural parameters, that is, not all combinations will ensure exceptional band-pass filters. For more details about the impact of the structural parameters on the band-pass filtering characteristics see the Supplementary Material.

Thirdly, we found that Gaussian gapped GSLs are the best option as band-pass filters when the number of barriers is reduced. In particular, we assessed the case of *N* = 9. However, it is expected that a critical *N* exists because for a very reduced number of barriers the profiles will be practically the same. According to our calculations Gaussian gapped GSLs are the best option up to *N* = 6, see the Supplementary Material. Here, it is important to take into account that when dealing with few barriers the stop and pass bands are not well-defined. So, as stop bands are as important as pass bands it is crucial to choose a considerable number of barriers that ensures good device functionality.

In fourth place, for gated GSLs we have considered potential barriers for electrons (positive *V*_*i*_’s) as well as electrons as the dominating charge carriers (positive bottom-gate voltage). However, the same results can be obtained if we consider barriers for holes (negative *V*_*i*_’s) and holes as the dominating charge carriers (negative bottom-gate voltage). The corresponding results can be found in the Supplementary Material. In the case of gapped GSLs the same potential profile corresponds to electrons and holes, see Fig. 1d. So, we can obtain essentially the same results for electrons or holes as dominating charge carriers. The results for holes are presented in the Supplementary Material.

In fifth place, it is important to remark that in the last part of the present study we focus our attention to Gaussian GSLs due to the relevance of this profile, however similar results can be obtained for the other non-conventional profiles. The transmission maps for the Lorentzian, Linear and Pöschl-Teller profiles are shown in the Supplementary Material.

Last but not least, it is the possible experimental device for non-conventional gapped GSLs. Although, heterostructured substrates are a possibility as far as we know there are not reports about this option. A more realistic possibility could be hydrogenated graphene. It is well known that a bandgap can be induced in graphene by patterned hydrogen adsorption^40^ as well as a reversible and tunable bandgap by varying the hydrogen coverage^41^. In addition, hydrogenated graphene can be used as active precursor to create hybrid superlattices^42^. So, in principle, graphene with regions with different hydrogen coverage (hydrogenated GSLs) could be a possible route to achieve non-conventional gapped GSLs, and hence efficient band-pass filters. Another possibility that could be reliable from the experimental standpoint is graphene over a uniform substrate such as SiC or hBN, and top gates and a back gate^43^. The uniform substrate opens a bandgap over the whole graphene sheet, while the top gates and the back gate generate the potential barriers and modulate the Fermi energy, respectively. The non-conventional potential profiles could be achievable, without the need of heterostructured substrates, by simply varying the top gate voltages in non-uniform fashion. This superlattice structure represents a mixed case of the gated and gapped GSLs that we are dealing with, and consequently a thorough study and analysis is needed in order to unveil the particularities that both cases in conjunction provide. Other 2D materials like bilayer graphene, silicene, phosphorene and transition-metal-dichalcogenides could be an option for electron band-pass filtering due to their intrinsic band structure characteristics. In particular, bilayer graphene represents an excellent option because a bandgap and the chirality of the charge carriers can be modulated with gating^44,45^.

## Conclusions

In summary, we show that the chiral nature of the charge carriers in graphene as well as the superlattice potential profile are essential for good band-pass filtering. By comparing the transmission properties of gated and gapped GSLs of Gaussian, Lorentzian, Linear and Pöschl-Teller potential profiles we obtain that the hole states inside the barriers of gated GSLs are the main obstacle for good band-pass filtering. Regardless of the potential profile and the number of barriers persistent oscillations in the transmission bands hamper the formation of perfect or nearly perfect pass bands. For gapped GSLs we obtain excellent band-pass filtering characteristics. Gaussian GSLs have the best filtering when the number of barriers is reduced, while all gapped GSLs are good filters, practically equivalent, for large number of barriers. Furthermore, we find that both gated and gapped non-conventional GSLs can work as omnidirectional band-pass filters. For gated GSLs it is important that the omnidirectional energy bandwidth does not include remanent oscillations. We consider that our results can be useful in designing electron band-pass filters based on non-conventional GSLs.

## Supplementary information

Supplementary Material
