# Supplementary material for: Non-conventional graphene superlattices as electron band-pass filters
Source: Sci Rep. 2019 Jun 19;9:8759. doi: 10.1038/s41598-019-45417-3 (PMC6584528; doi:10.1038/s41598-019-45417-3)
Supplement: Supplementary file 1 — Supplementary Material [file 41598_2019_45417_MOESM1_ESM.pdf]

Supplementary Information

Non-conventional graphene superlattices as electron band-pass filters

A. Sánchez-Arellano[1], J. Madrigal-Melchor[1,2] and I. Rodríguez-Vargas[1,2]

[1] *Unidad Académica de Física, Universidad Autónoma de Zacatecas, Calzada Solidaridad Esquina Con Paseo La Bufa S/N, 98060 Zacatecas, Zac., México.*

[2] *Unidad Académica de Ciencia y Tecnología de la Luz y la Materia, Universidad Autónoma de Zacatecas, Carretera Zacatecas-Guadalajara Km. 6, Ejido La Escondida, 98160 Zacatecas, Zac., México.*

S.I Impact of the structural parameters on the band-pass filtering characteristics of non-conventional GSLs

S.I.1 Variation of the width/spacing of the barriers

Here, we show the results of non-conventional gapped and gated GSLs for different values of the width/spacing of the barriers. It is important to highlight that the total length of the superlattice structure is the same in all cases.

Regardless of the width/spacing of the barriers, the Gaussian gapped GSLs are the best option for band-pass filtering. However, as the width/spacing of the barriers is increased even the pass bands of the gapped Gaussian profile present oscillations. The flatness of the pass bands can be recovered by adjusting the superlattice structural parameters, in particular the maximum height of the barriers. Check the corresponding results in the following subsection.

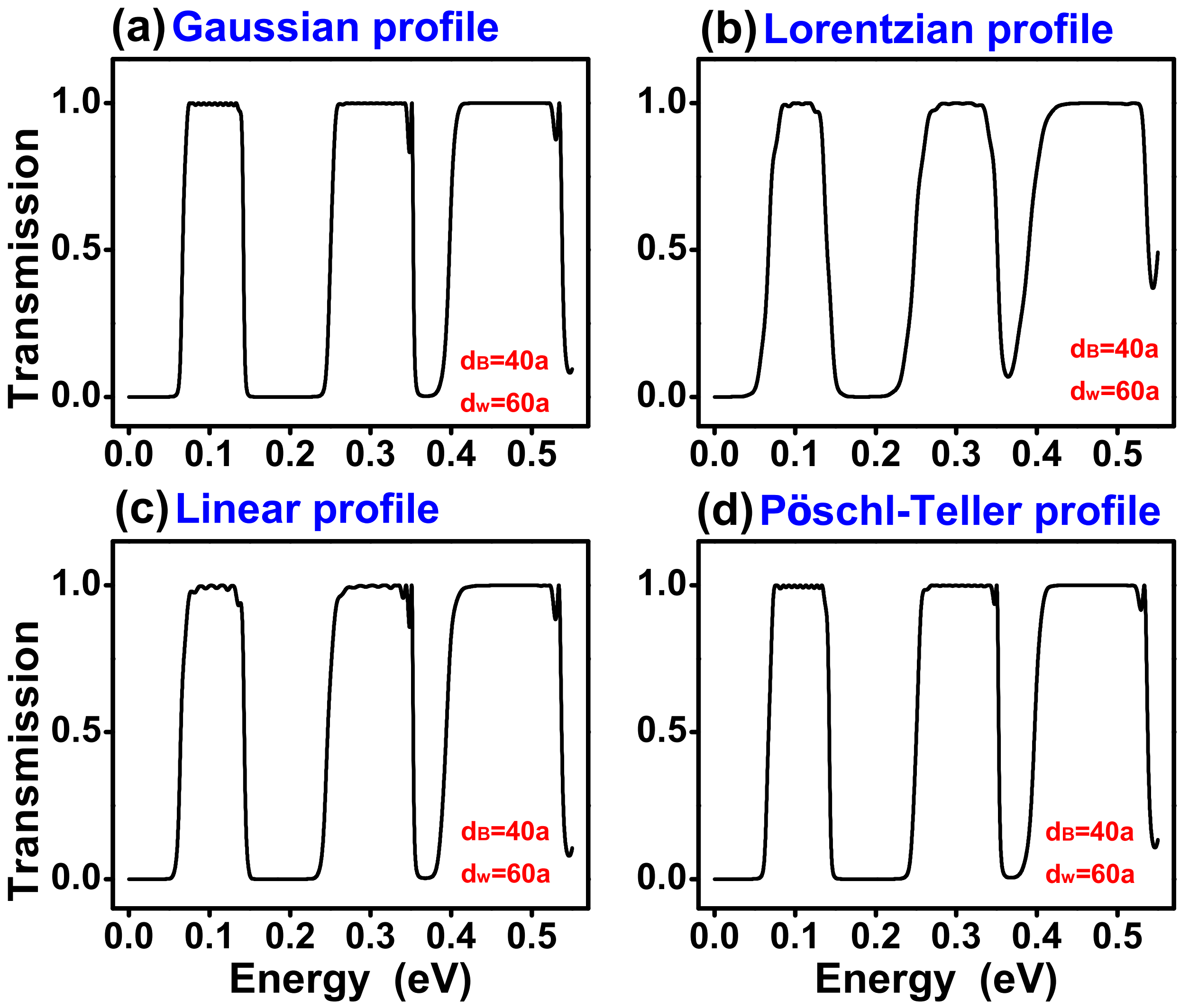

Fig. S1: Transmittance versus the energy of electrons of gapped GSLs with (a) Gaussian, (b) Lorentzian, (c) Linear and (d) Pöschl-Teller potential profiles for a width/spacing (d_B/d_W) of the barriers of $40a/60a$. The other structural parameters are: $t'_{max} = 0.13$ eV, $t'_{min} = 0.01$ eV, $\theta = 45°$ and $N = 21$.

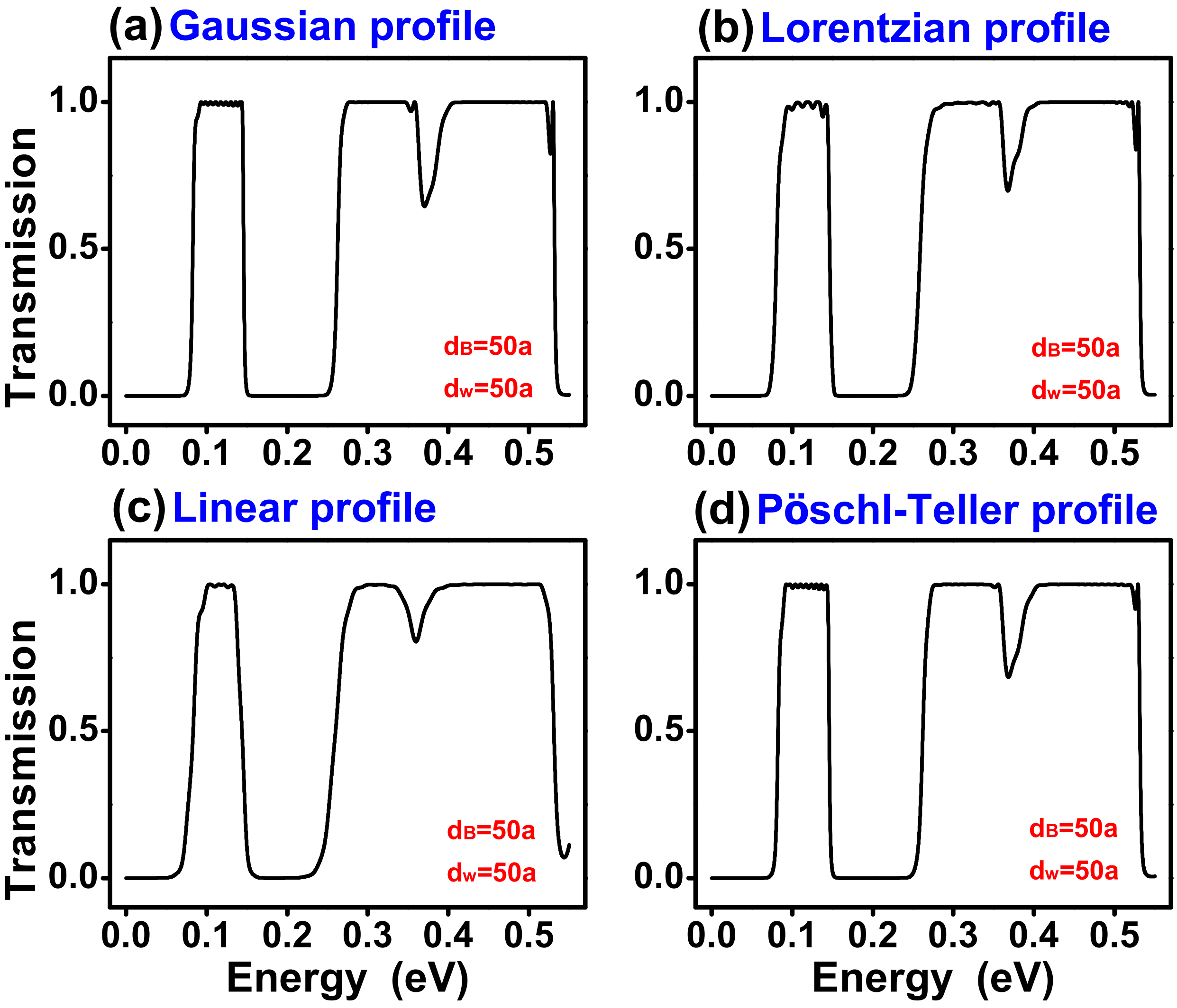

Fig. S2: The same as in Fig. S1, but here the width/spacing of the barriers is $50a/50a$.

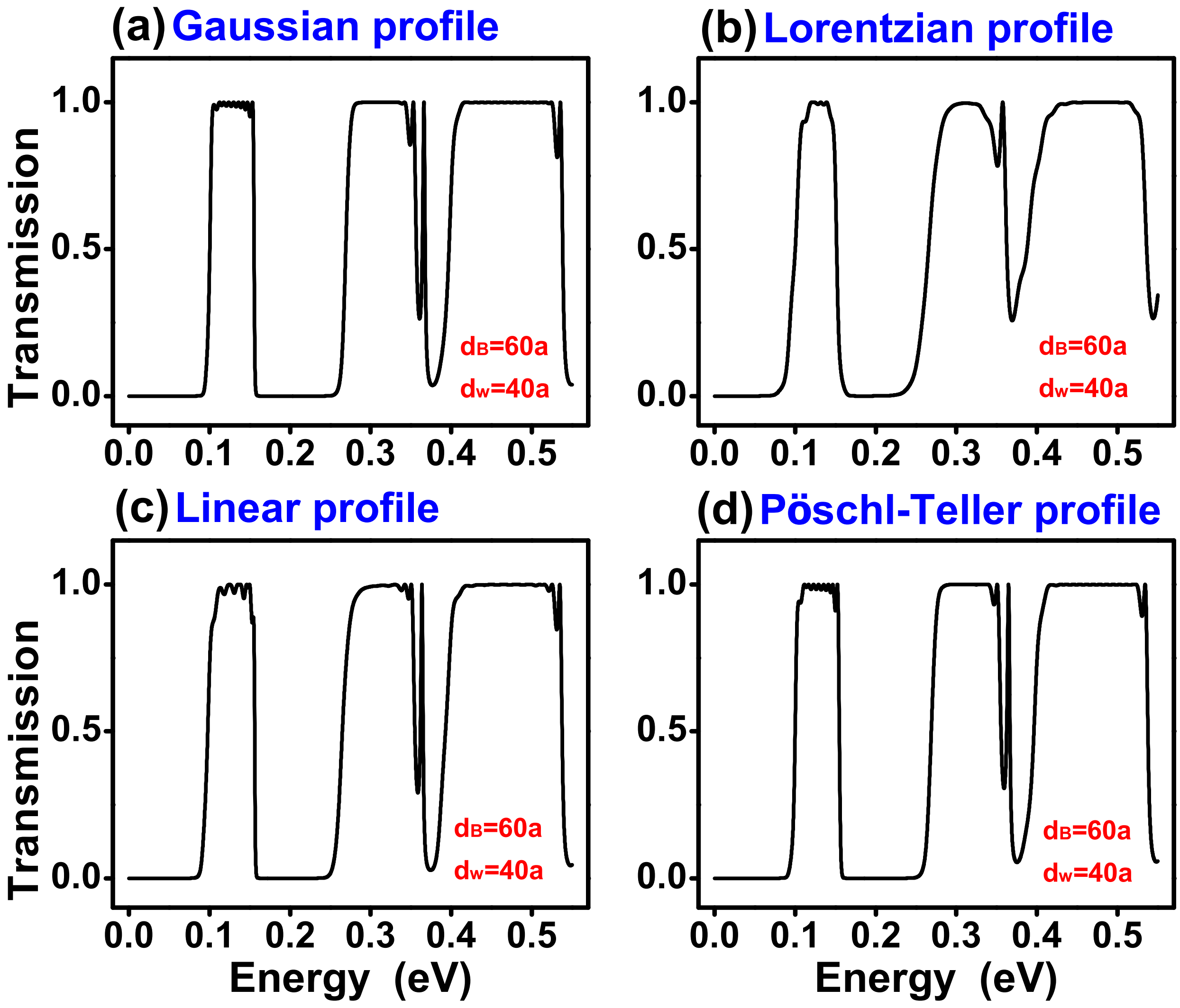

Fig. S3: The same as in Fig. S1 and S2, but here the width/spacing of the barriers is $60a/40a$.

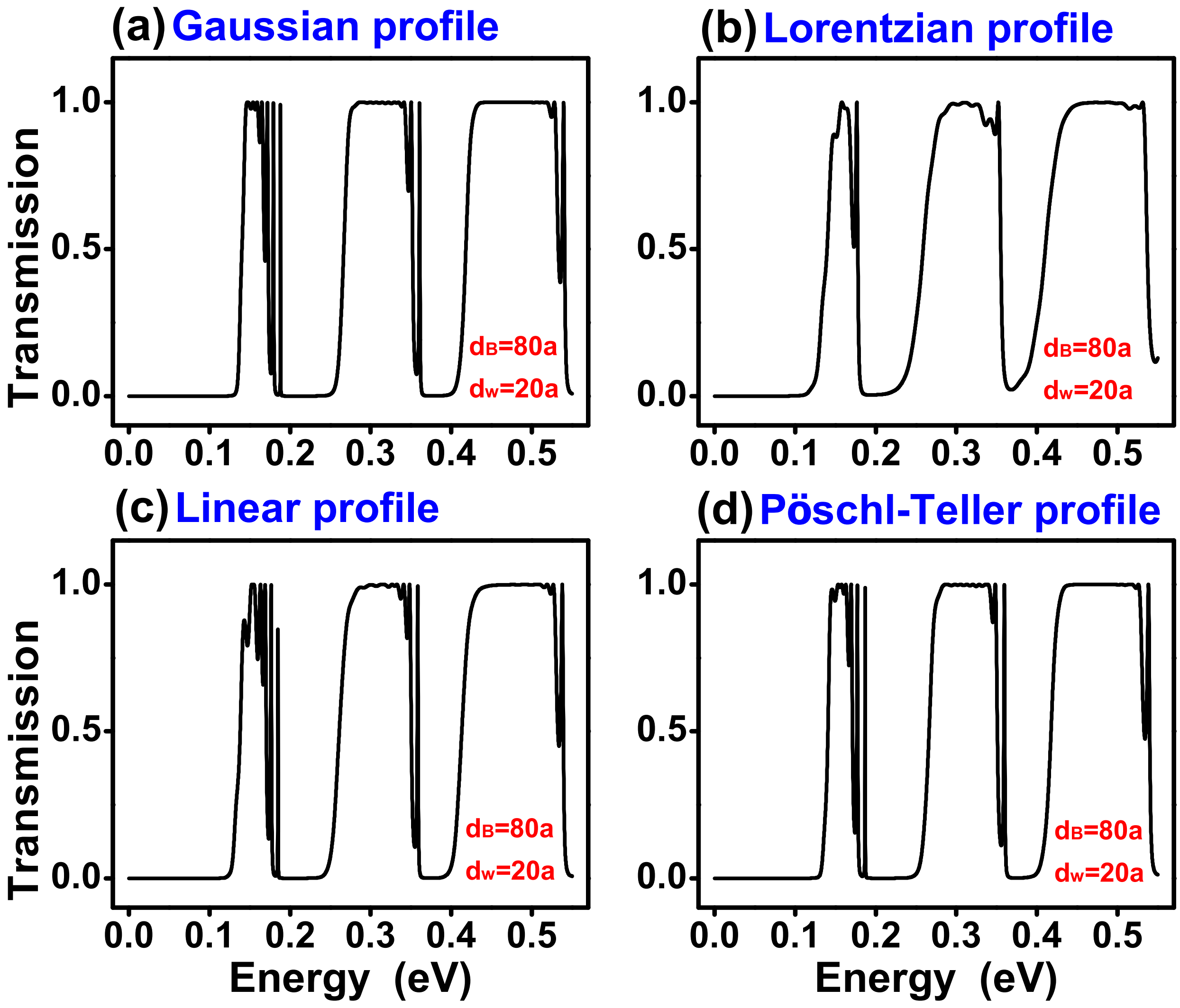

Fig. S4: The same as in Fig. S1, S2 and S3, but here the width/spacing of the barriers is $80a/20a$.

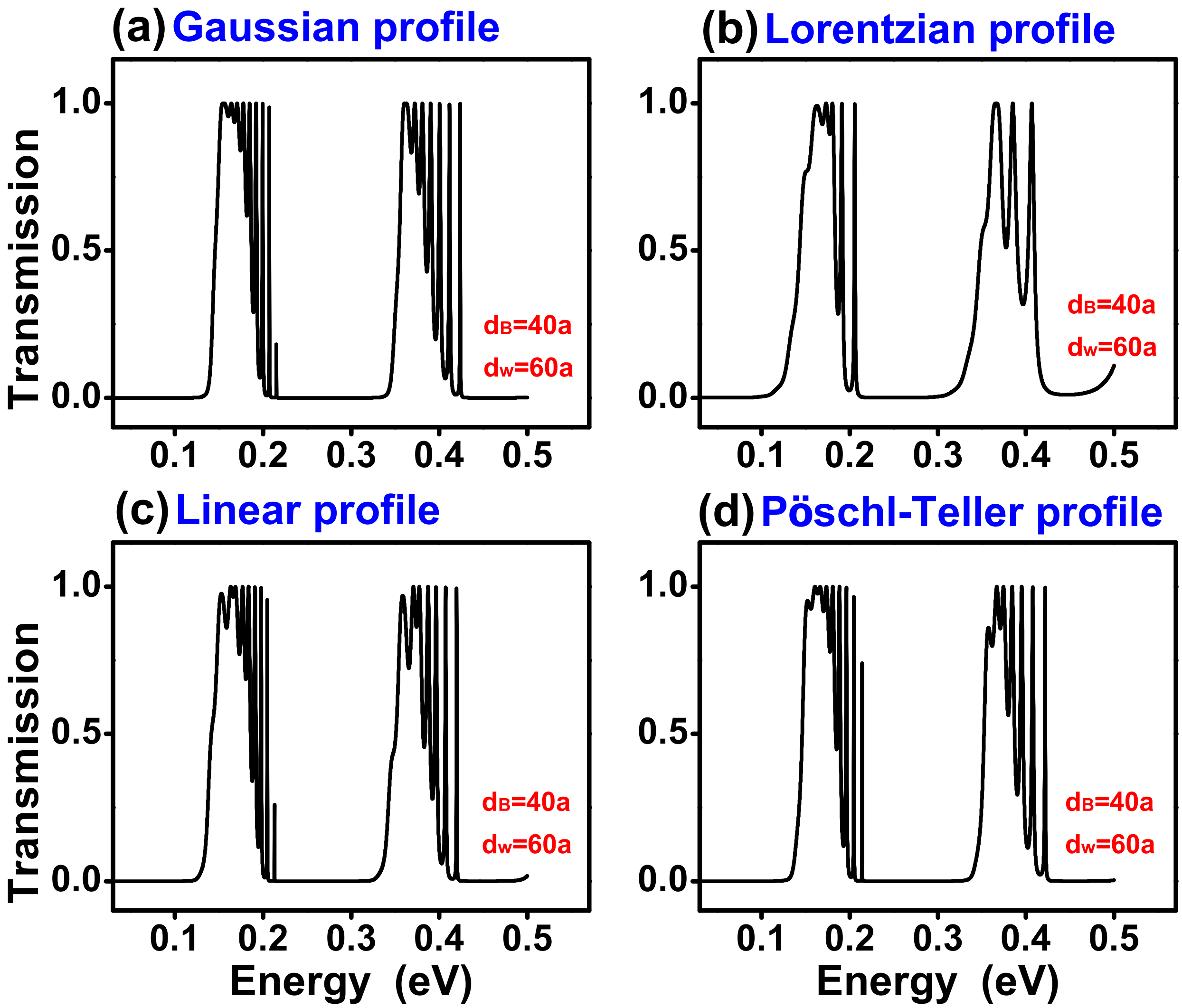

Fig. S5: Transmittance versus the energy of electrons of gated GSLs with (a) Gaussian, (b) Lorentzian, (c) Linear and (d) Pöschl-Teller potential profiles for a width/spacing (d_B/d_W) of the barriers of $40a/60a$. The other structural parameters are: $V_{max} = 0.13$ eV, $V_{min} = 0.01$ eV, $\theta = 45°$ and $N = 21$.

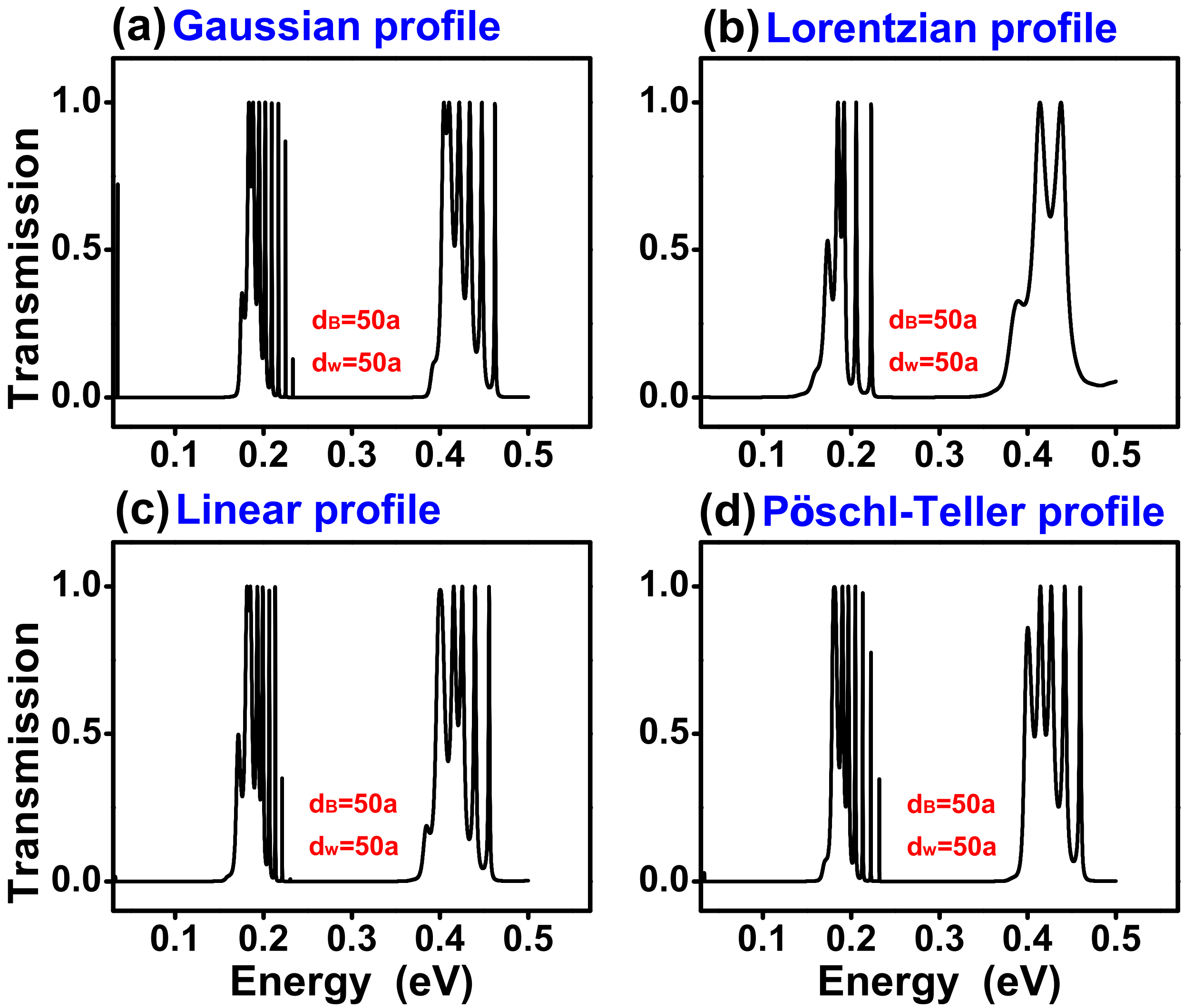

Fig. S6: The same as in Fig. S5, but here the width/spacing of the barriers is $50a/50a$.

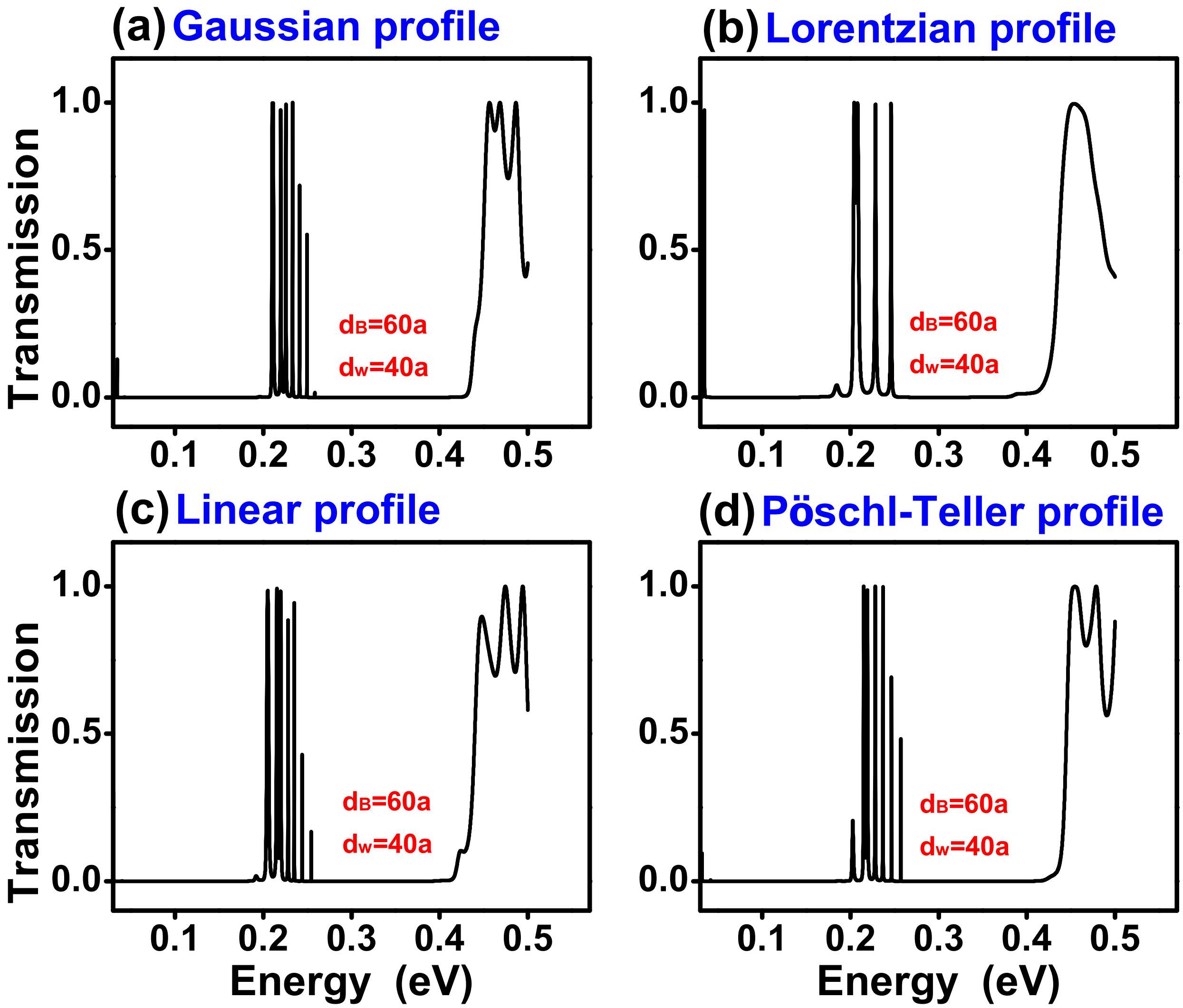

Fig. S7: The same as in Fig. S5 and S6, but here the width/spacing of the barriers is $60a/40a$.

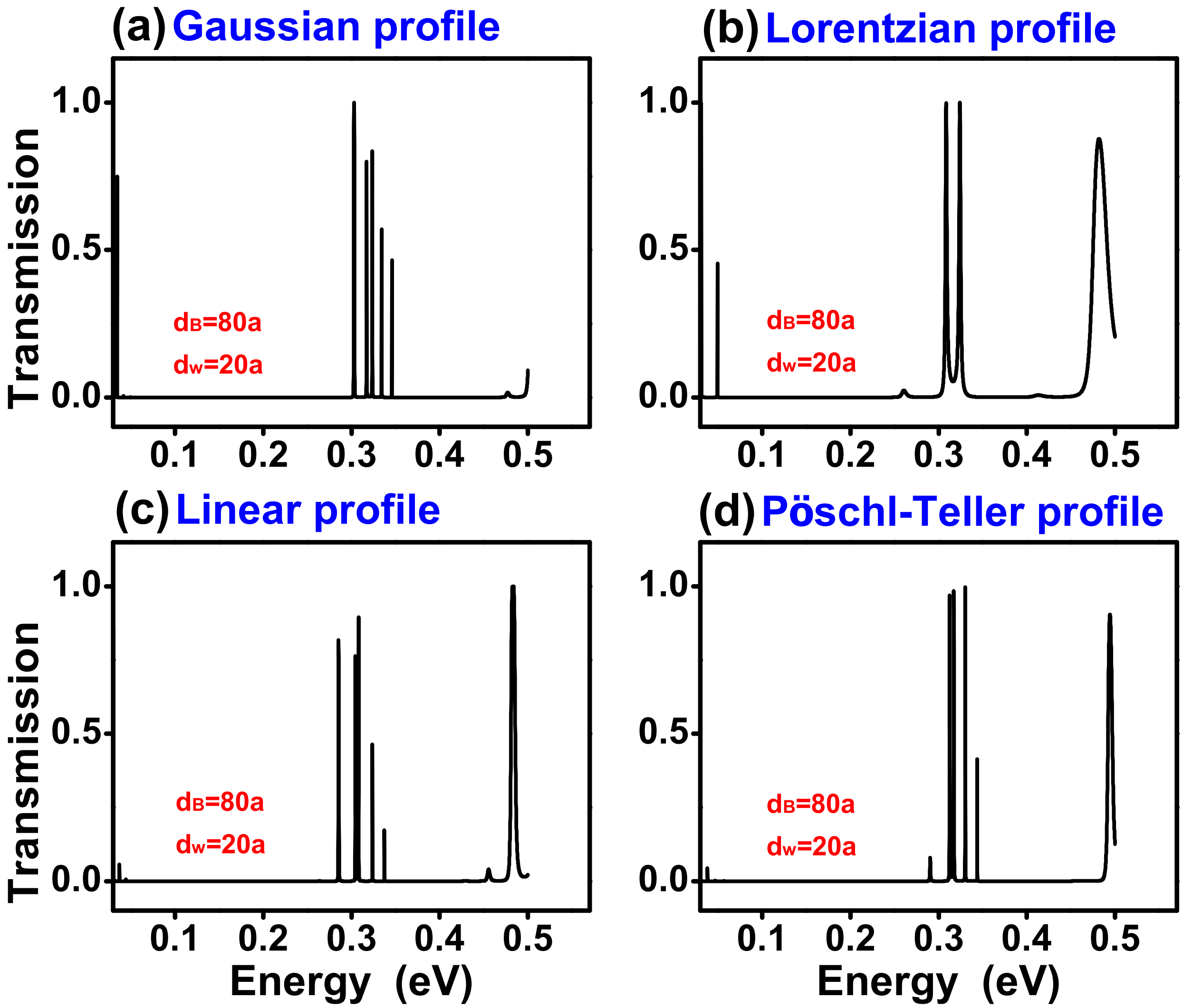

Fig. S8: The same as in Fig. S5, S6 and S7, but here the width/spacing of the barriers is $80a/20a$.

S.I.2 Variation of the maximum barrier height

By adjusting the maximum barrier height of non-conventional GSLs it is possible to improve or worsen the flatness of the pass bands. Here, we have chosen the case $80a/20a$ of width/spacing of the barriers for the gapped GSLs of the preceding section. By lowering t'_{max} it is possible to improve the flatness of the pass bands. On the contrary, by increasing it the flatness is worsen.

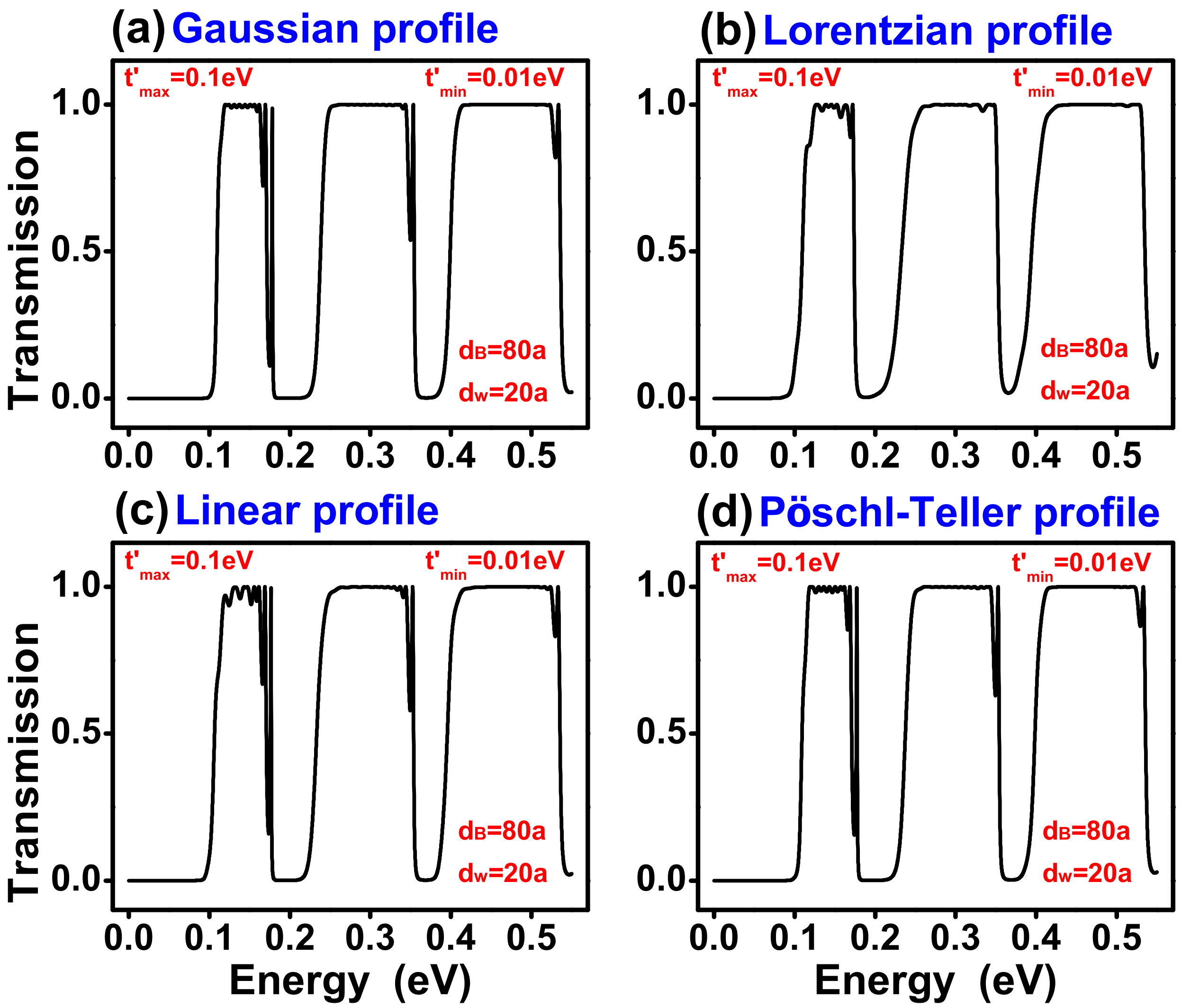

Fig. S9: Transmittance versus the energy of electrons of gapped GSLs with (a) Gaussian, (b) Lorentzian, (c) Linear and (d) Pöschl-Teller potential profiles for a maximum barrier height $t'_{max} = 0.1$ eV. The other structural parameters are: $t'_{min} = 0.01$ eV, $d_B = 80a$, $d_W = 20a$, $\theta = 45°$ and $N = 21$.

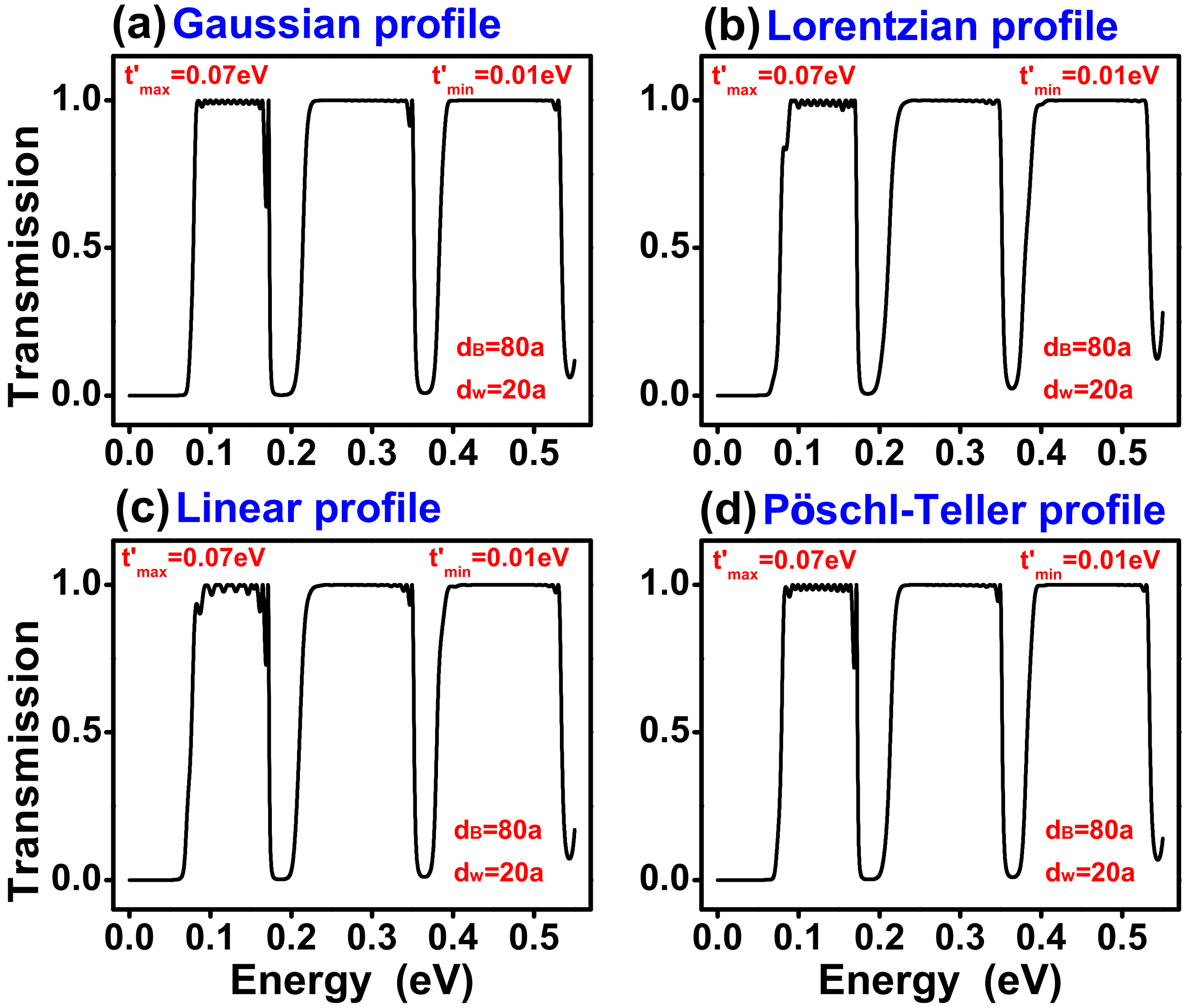

Fig. S10: The same as in Fig. S9, but here $t'_{max} = 0.7$ eV.

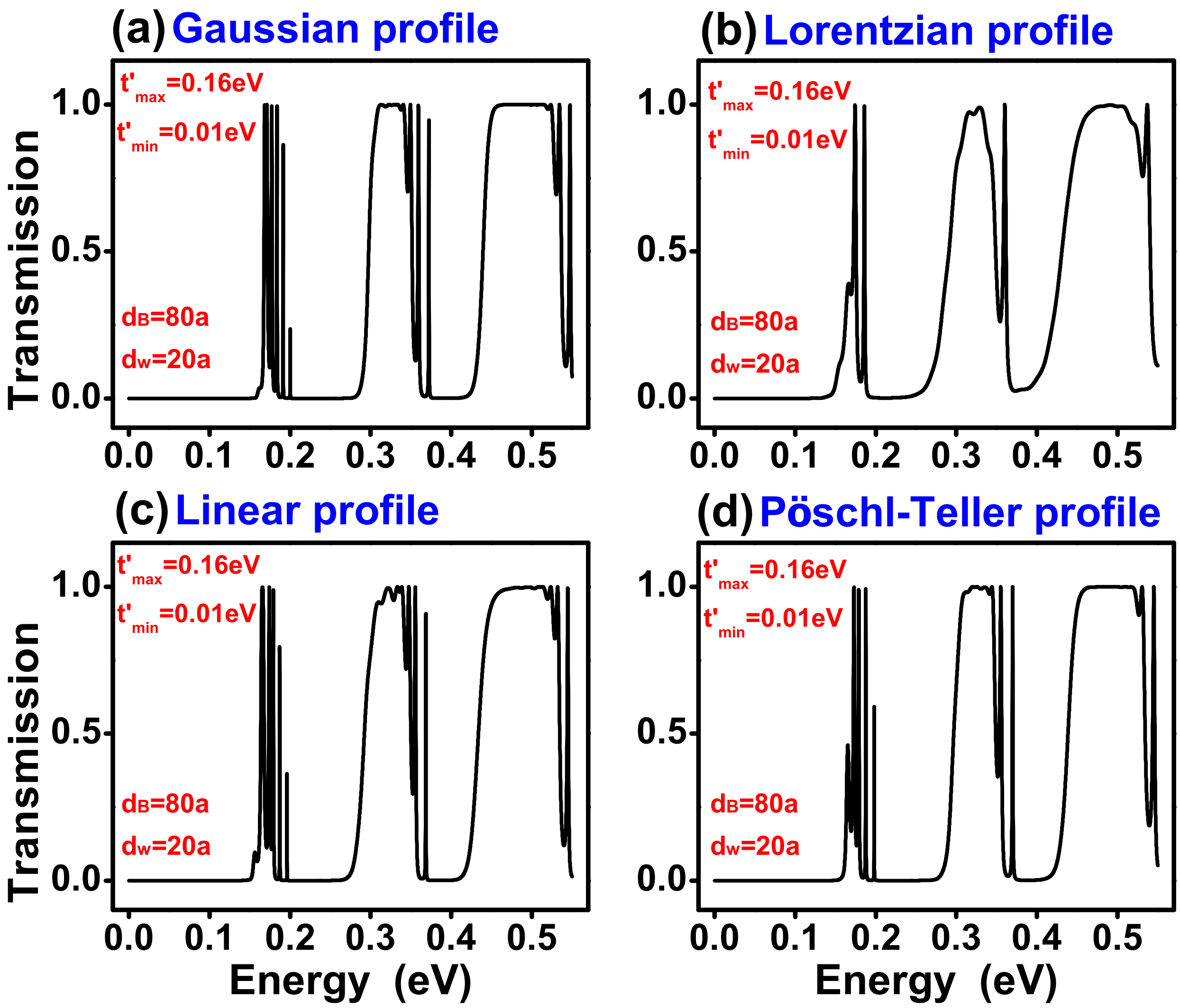

Fig. S11: The same as in Fig. S9 and S10, but here $t'_{max} = 0.16$ eV.

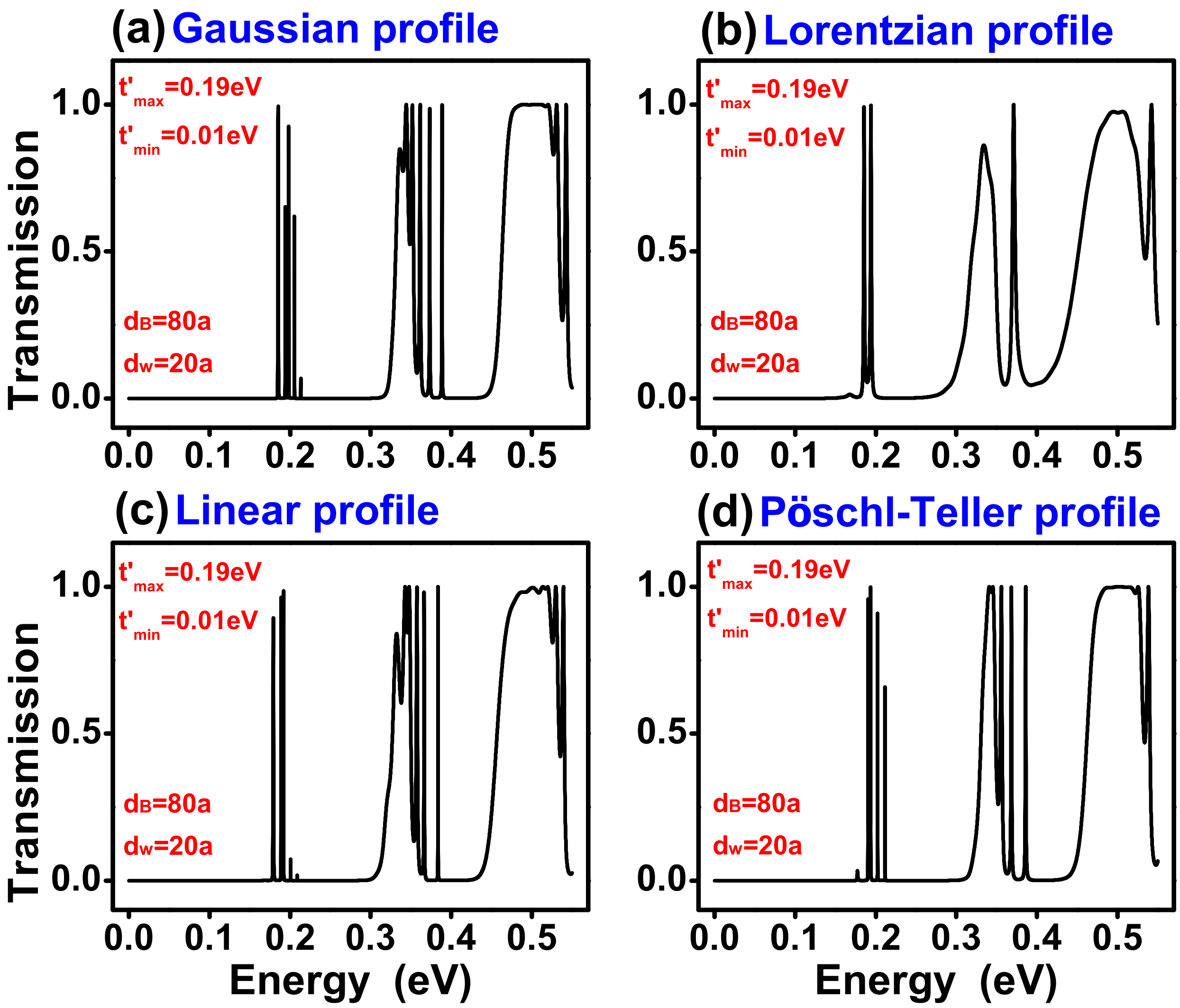

Fig. S12: The same as in Fig. S9, S10 and S11, but here $t'_{max} = 0.19$ eV.

S.I.3 Variation of the angle of incidence

The angle of incidence can also be a tuning parameter. Here, we show that by increasing or decreasing the angle of incidence it is possible to modulate the number and width of the pass bands of gapped and gated GSLs.

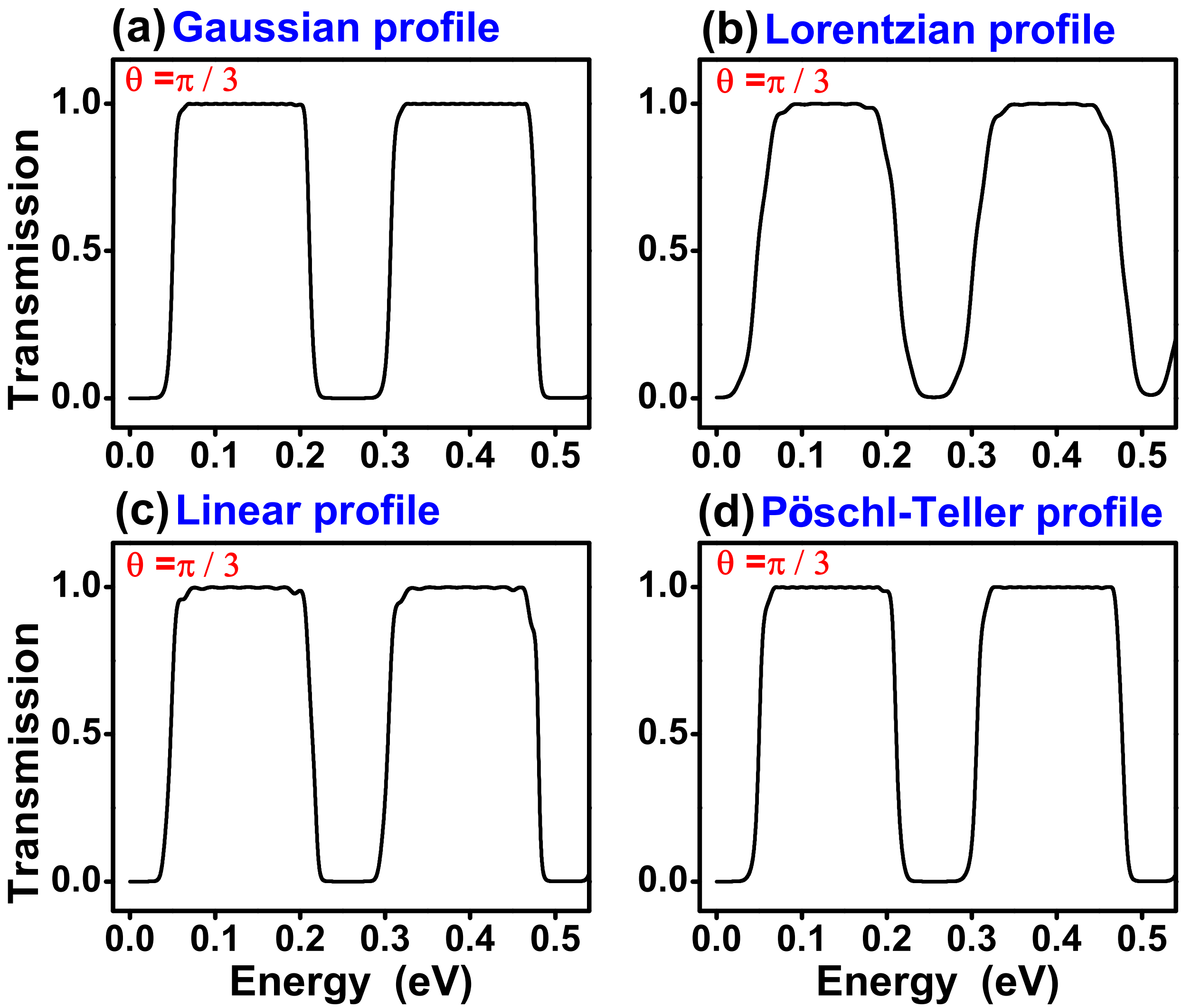

Fig. S13: Transmittance versus the energy of electrons of gapped GSLs with (a) Gaussian, (b) Lorentzian, (c) Linear and (d) Pöschl-Teller potential profiles for an angle of incidence $\theta = 60°$. The other structural parameters are: $t'_{max} = 0.13$ eV, $t'_{min} = 0.01$ eV, $d_B = 20a$, $d_W = 80a$ and $N = 21$.

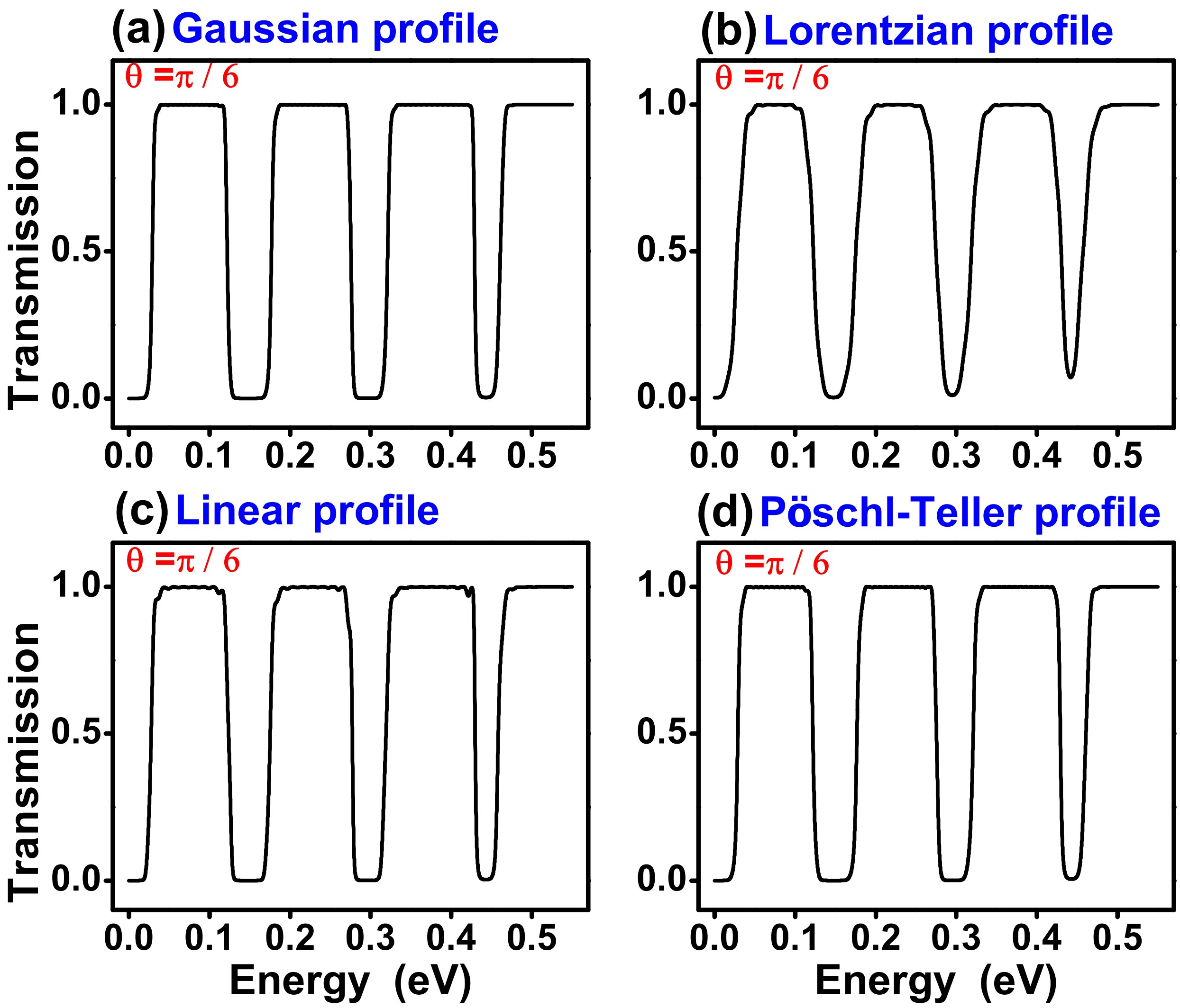

Fig. S14: The same as in Fig. S13, but in this case $\theta = 30^{\circ}$.

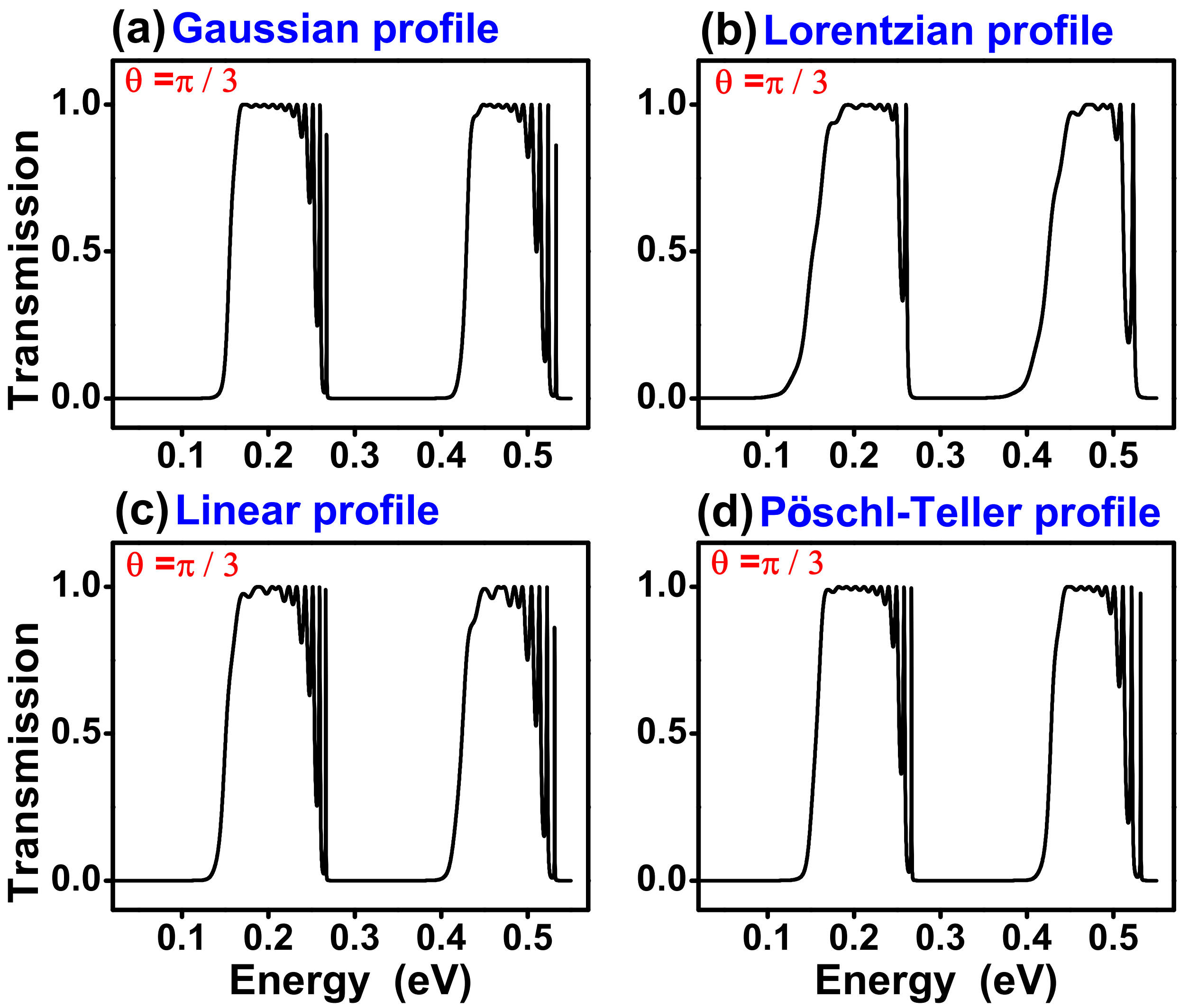

Fig. S15: The same as in Fig. S13, but for gated GSLs.

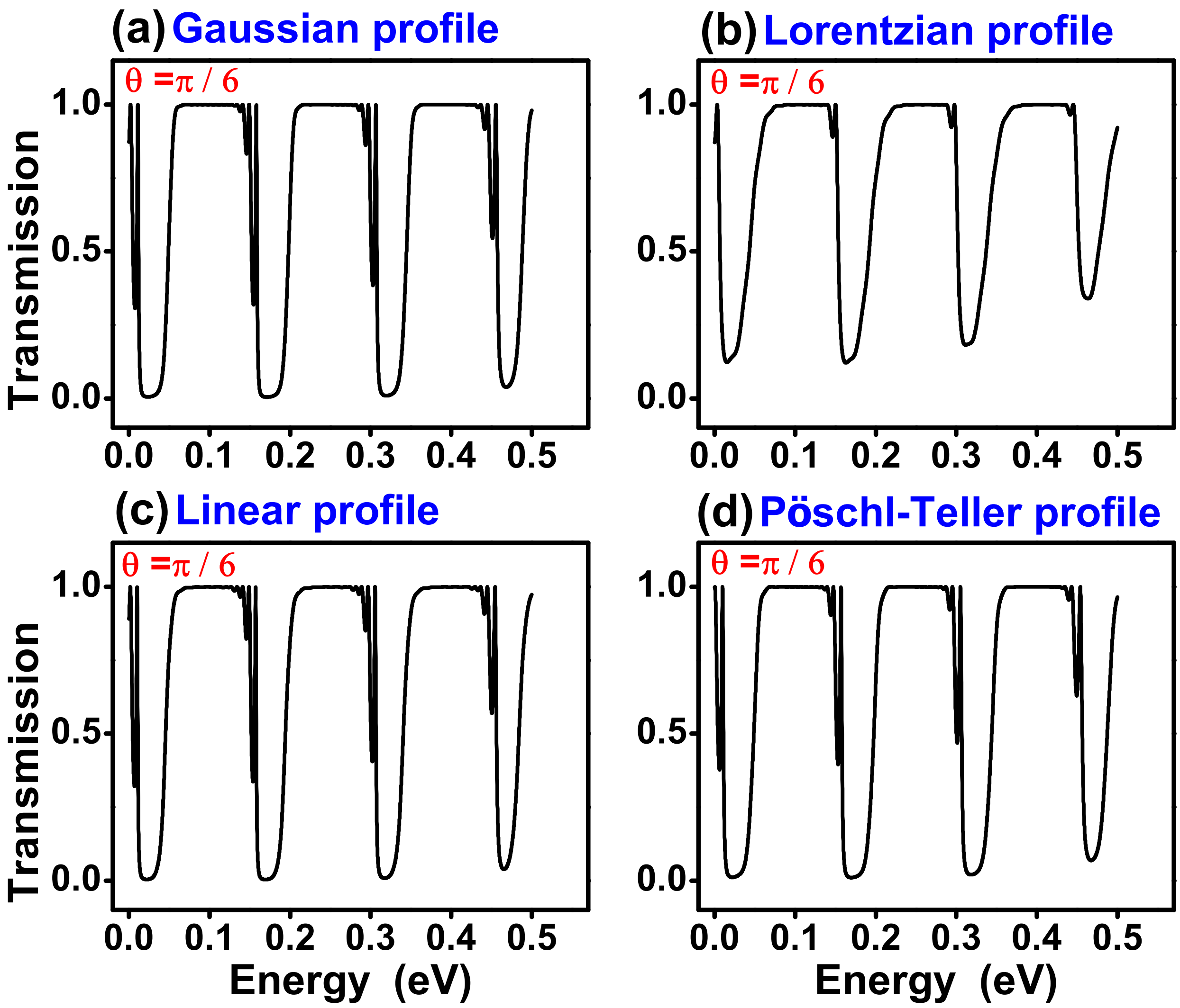

Fig. S16: The same as in Fig. S15, but here $\theta = 30°$.

S.II Band-pass filtering characteristics for $N < 9$

In this section we show the results of the band-pass filtering properties of gapped and gated GSLs for $N < 9$. We find that the gapped Gaussian profile is the best option up to $N = 6$.

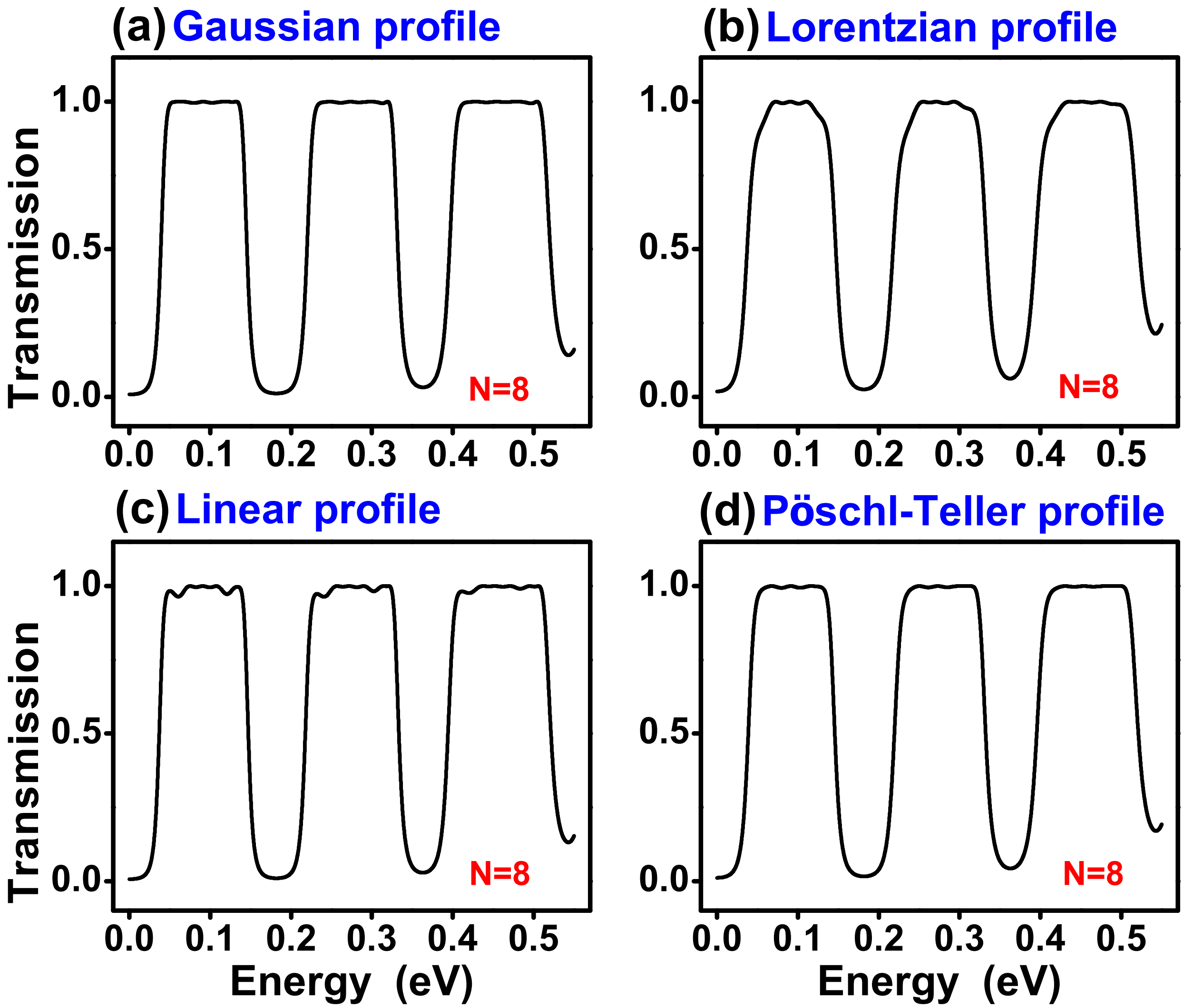

Fig. S17: Transmittance versus the energy of electrons of gapped GSLs with (a) Gaussian, (b) Lorentzian, (c) Linear and (d) Pöschl-Teller potential profiles for $N = 8$. The other structural parameters are: $t'_{max} = 0.13$ eV, $t'_{min} = 0.01$ eV, $d_B = 20a$, $d_W = 80a$ and $\theta = 45°$.

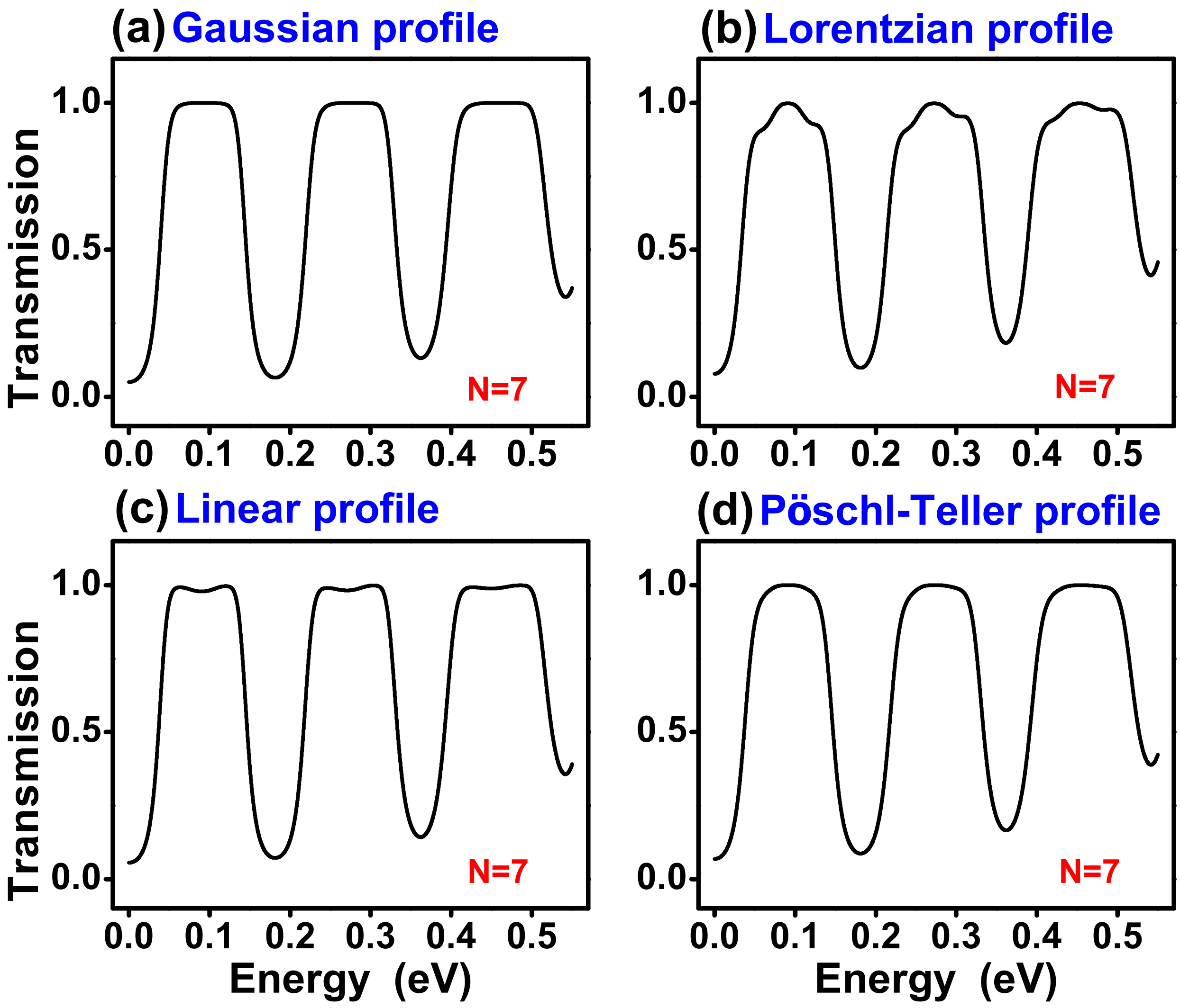

Fig. S18: The same as in Fig. S17, but in this case $N = 7$.

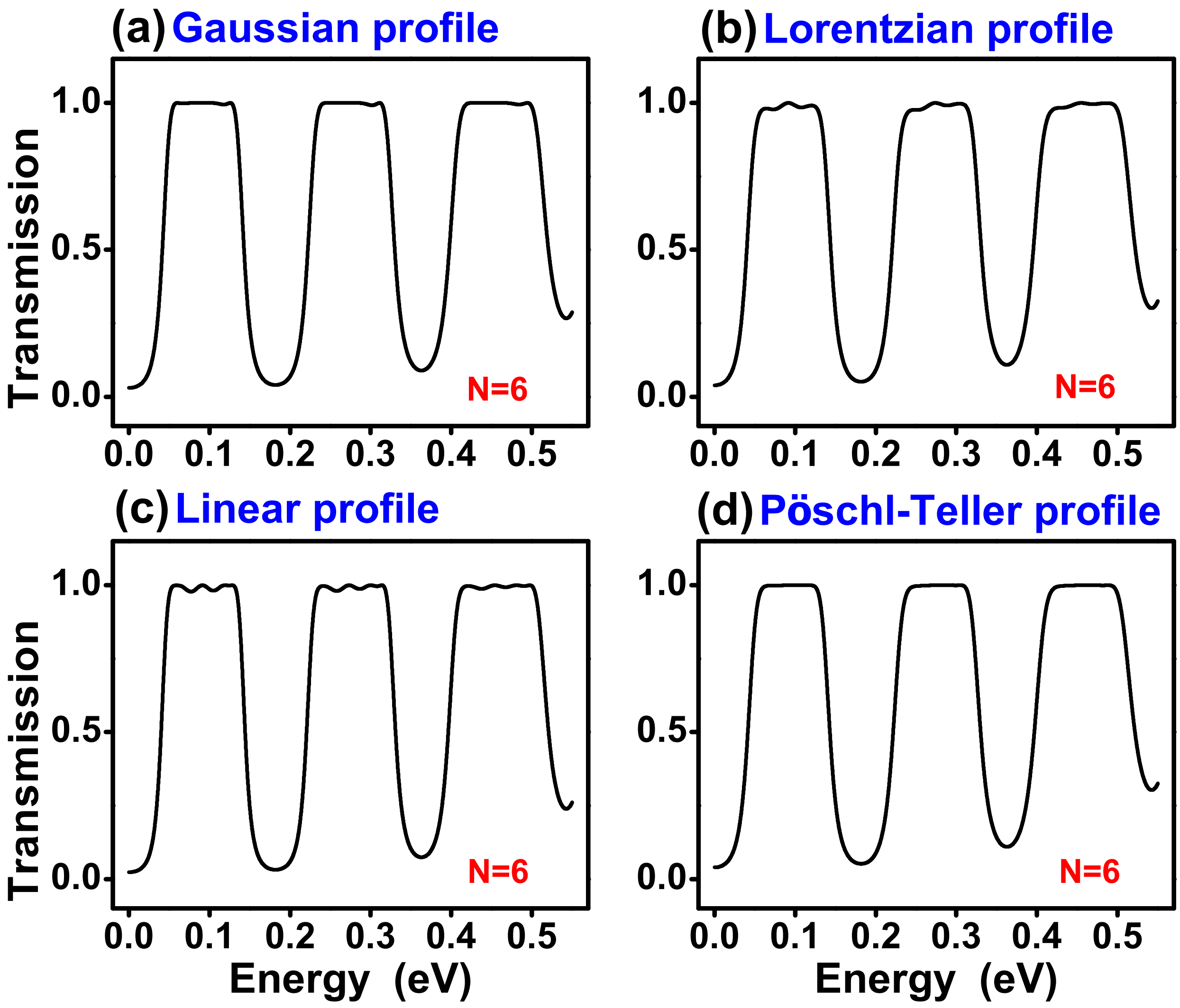

Fig. S19: The same as in Fig. S17 and S18, but in this case $N = 6$.

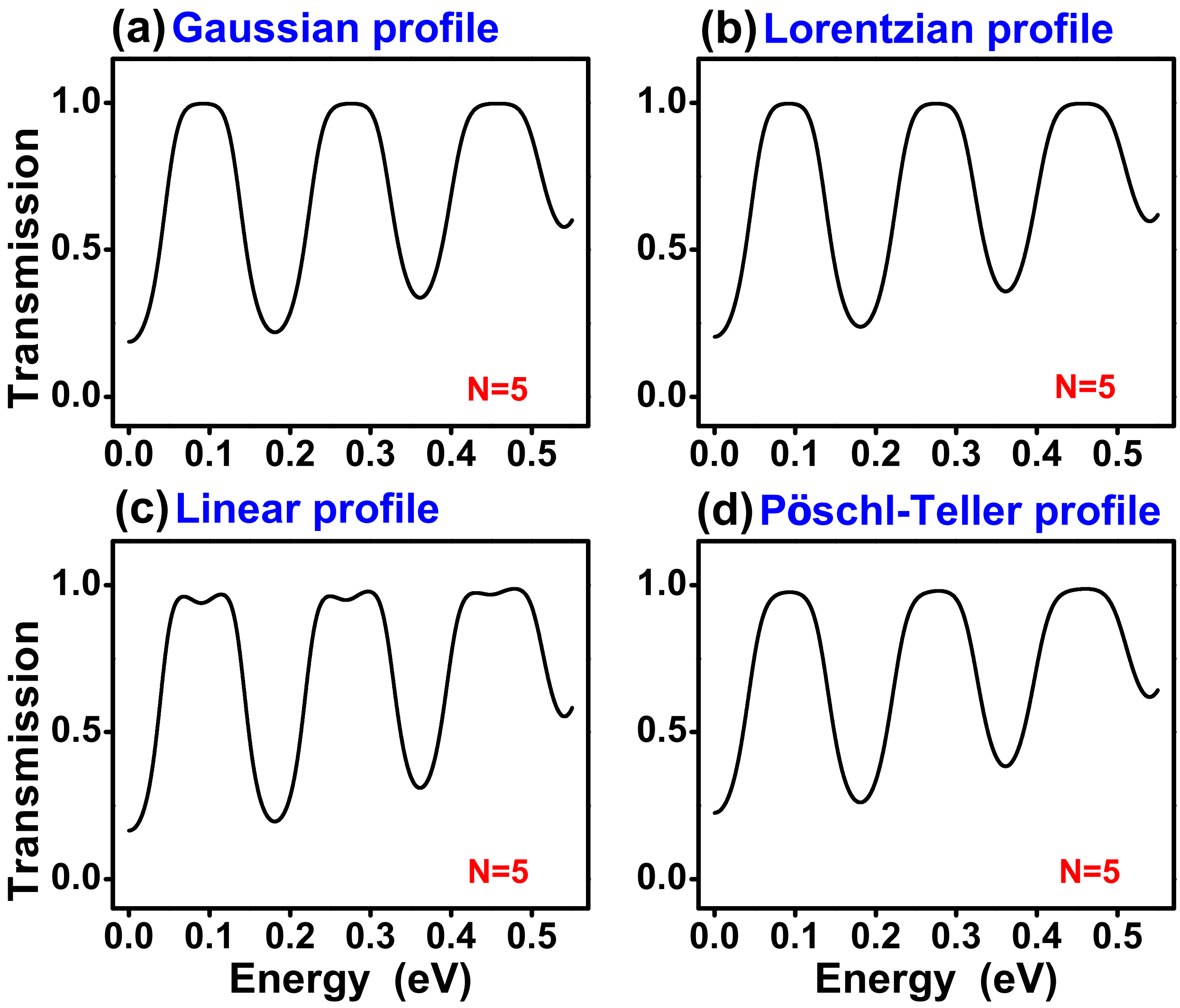

Fig. S20: The same as in Fig. S17, S18 and S19, but in this case $N = 5$.

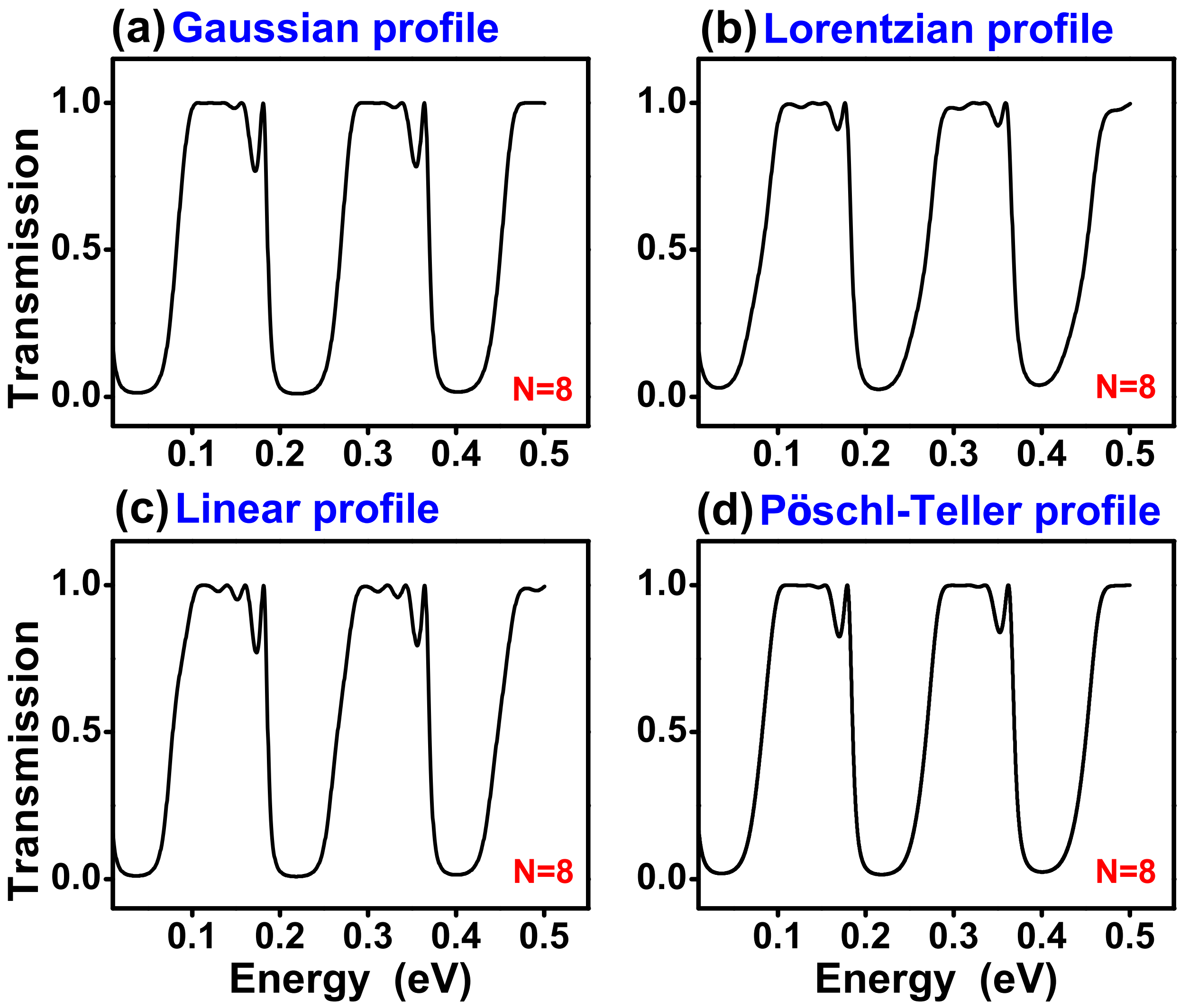

Fig. S21: Transmittance versus the energy of electrons of gated GSLs with (a) Gaussian, (b) Lorentzian, (c) Linear and (d) Pöschl-Teller potential profiles for $N = 8$. The other structural parameters are: $V_{max} = 0.13$ eV, $V_{min} = 0.01$ eV, $d_B = 20a$, $d_W = 80a$ and $\theta = 45°$.

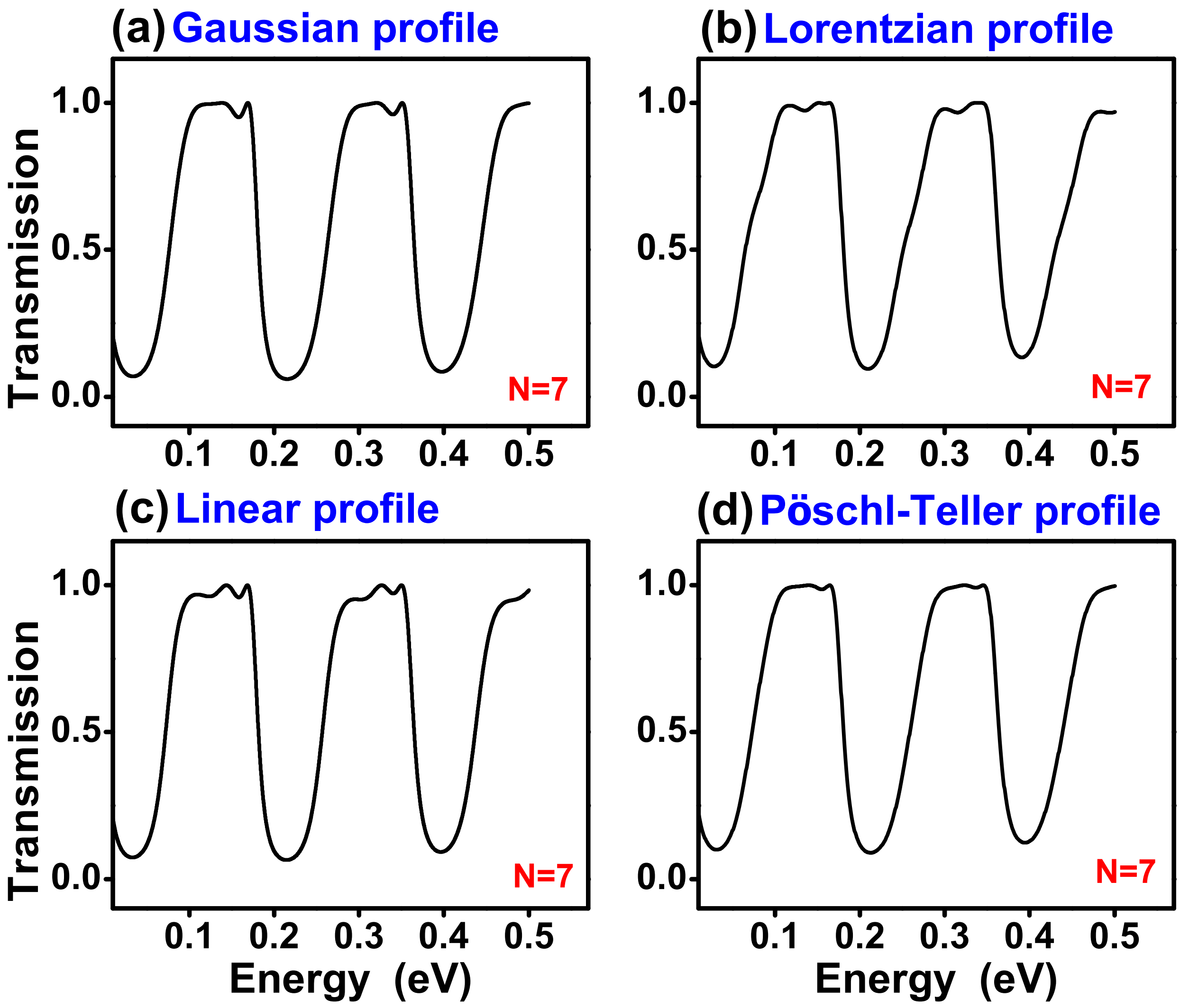

Fig. S22: The same as Fig. S21, but here $N = 7$.

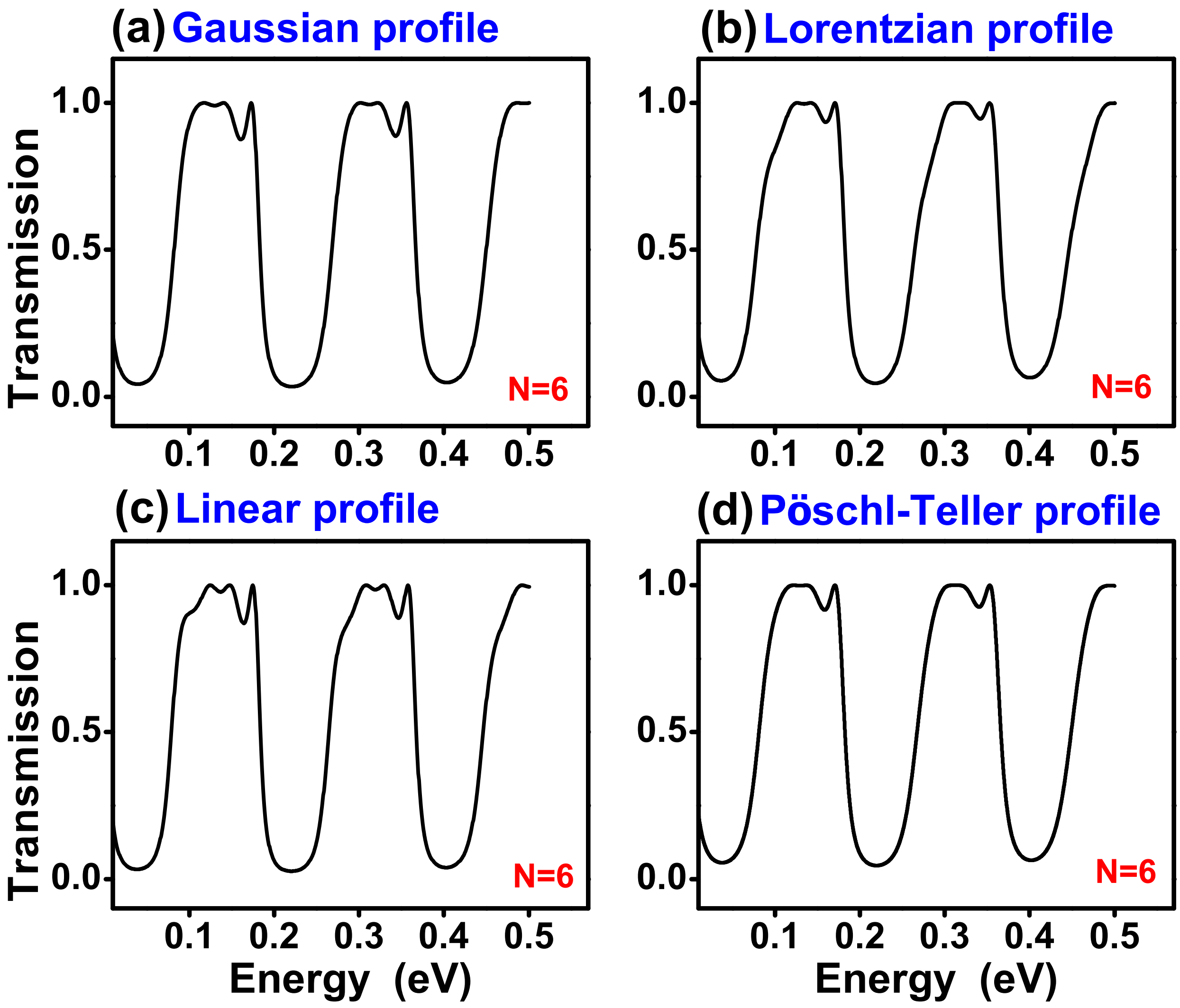

Fig. S23: The same as Fig. S21 and S22, but here $N = 6$.

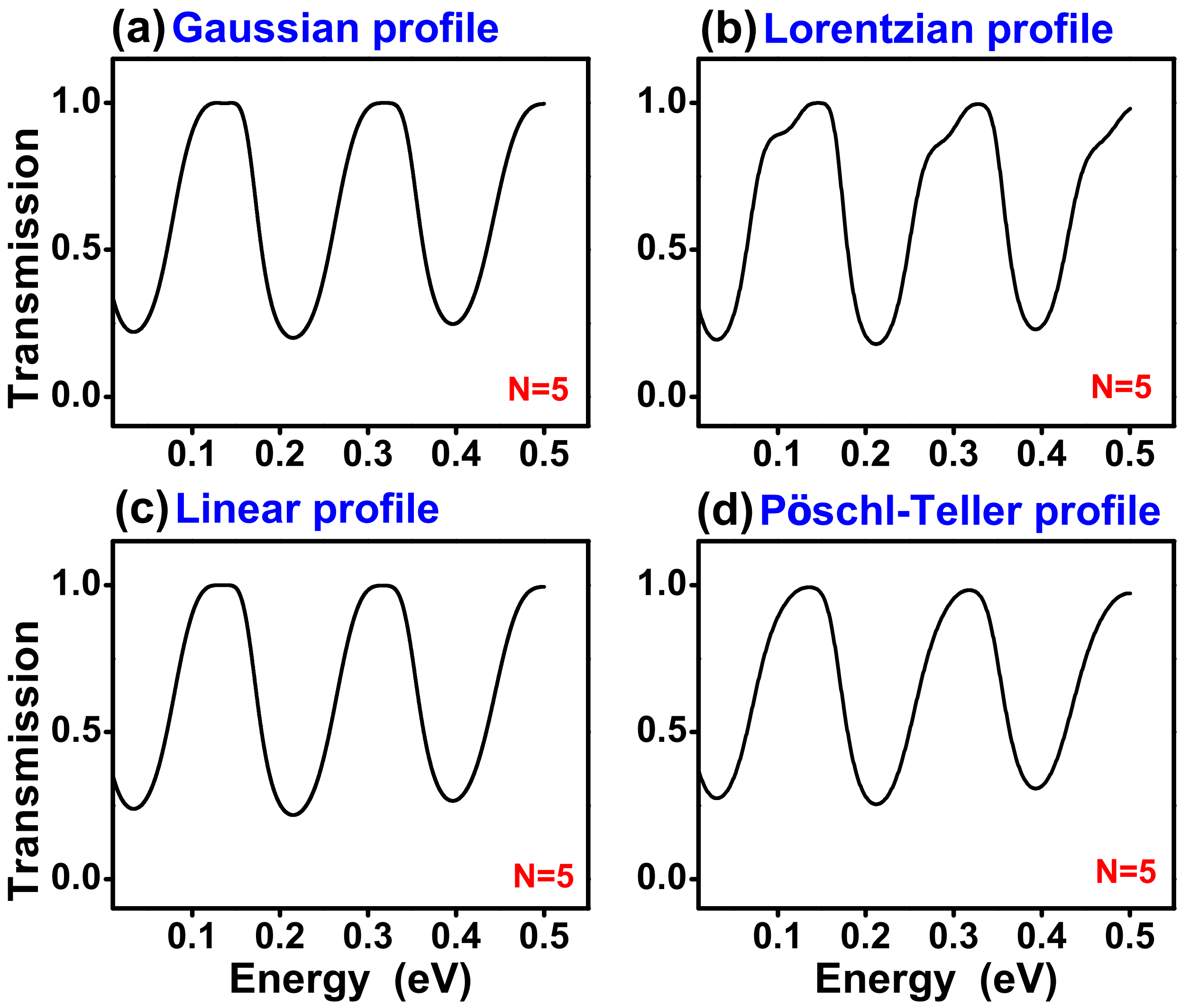

Fig. S24: The same as Fig. S21, S22 and S23, but here $N = 5$.

S.III Results when the holes are the dominating charge carriers

The same results for the band-pass filtering characteristics of non-conventional gated GSLs can be obtained if the potential barriers are negative (negative V_i's) and the dominating charge carriers are holes (negative bottom-gate voltage). In the case of non-conventional gapped GSLs we have the same potential profile for electrons and holes, see Fig. 1d in the manuscript. So, the band-pass filtering properties are the same when electrons and holes are

the dominating charge carriers, positive and negative bottom-gate voltage, respectively.

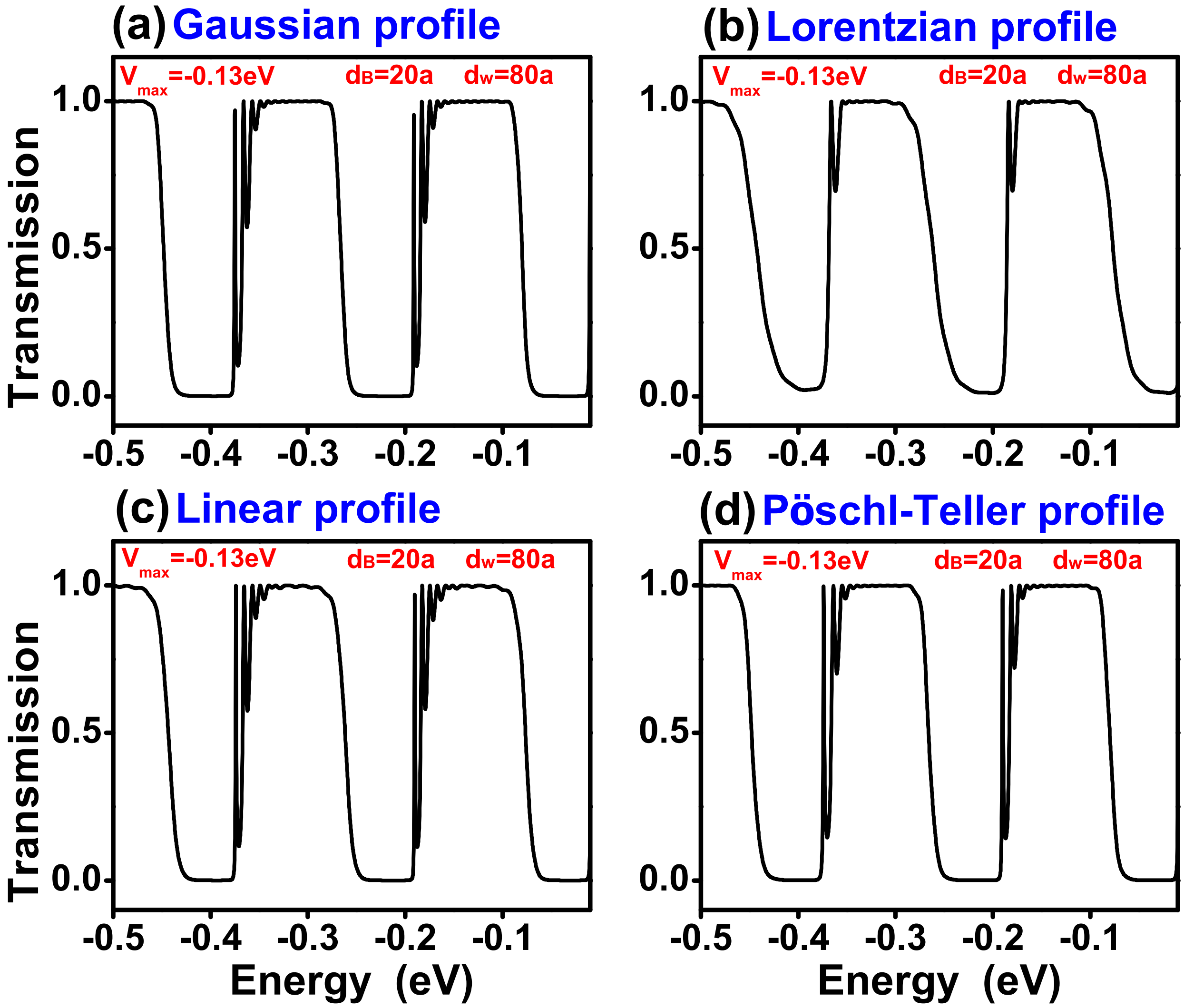

Fig. S25: Transmission properties of gated GSLs with (a) Gaussian, (b) Lorentzian, (c) Linear and (d) Pöschl-Teller potential profiles. In this case the potential barriers are negative (negative V_i's) and the dominating charge carriers are holes ($E < 0$). The superlattice parameters are: $V_{max} = -0.13$ eV, $V_{min} = -0.01$ eV, $d_B = 20a$, $d_W = 80a$, $\theta = 45°$ and $N = 21$.

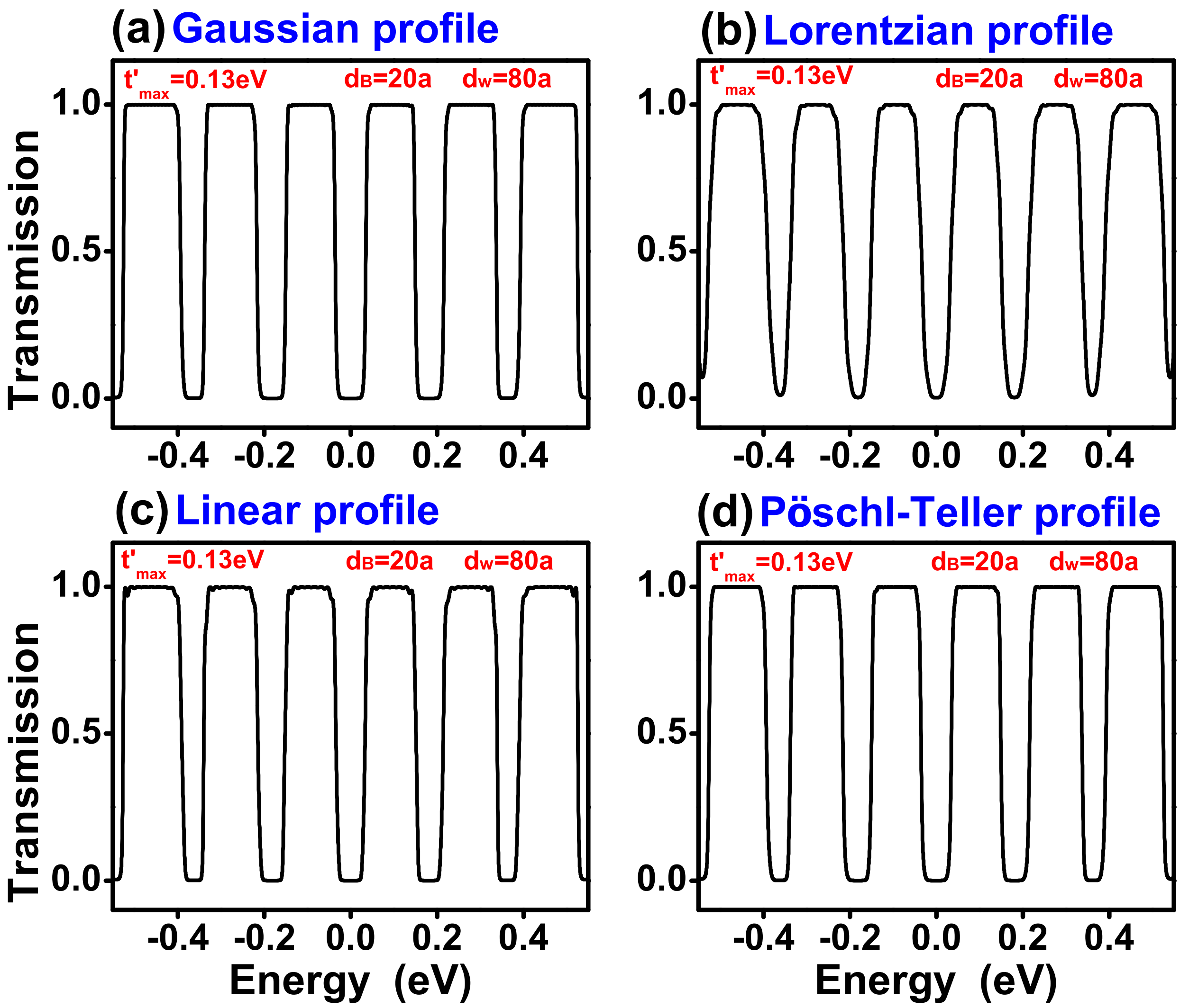

Fig. S26: Transmission properties of gapped GSLs with (a) Gaussian, (b) Lorentzian, (c) Linear and (d) Pöschl-Teller potential profiles. In this case, the same potential profile corresponds to electrons ($E > 0$) and holes ($E < 0$). Hence, the transmittance is symmetric with respect to $E = 0$. The superlattice parameters are: $t'_{max} = 0.13$ eV, $t'_{min} = 0.01$ eV, $d_B = 20a$, $d_W = 80a$, $\theta = 45°$ and $N = 21$.

S.IV Transmission maps for Lorentzian, Linear and Pöschl-Teller GSLs

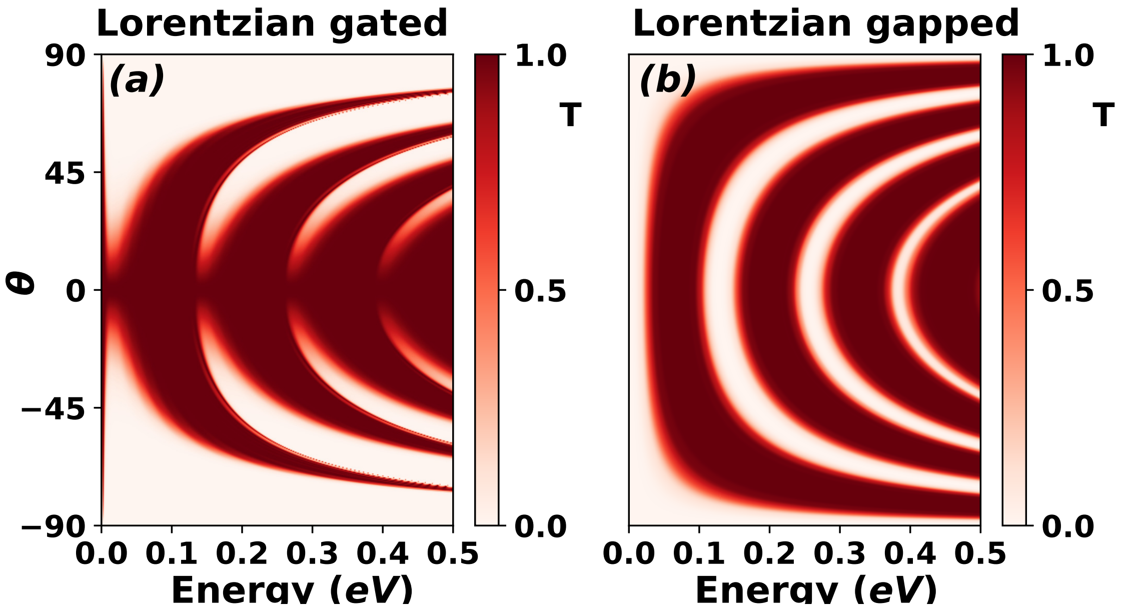

Fig. S27: Contour maps (E, θ) of the transmittance for (a) gated and (b) gapped Lorentzian GSLs. The superlattice parameters are: $V_{max} = t'_{max} = 0.13$ eV, $V_{min} = t'_{min} = 0.01$ eV, $d_B = 20a$, $d_W = 80a$, $\theta = 45°$ and $N = 21$.

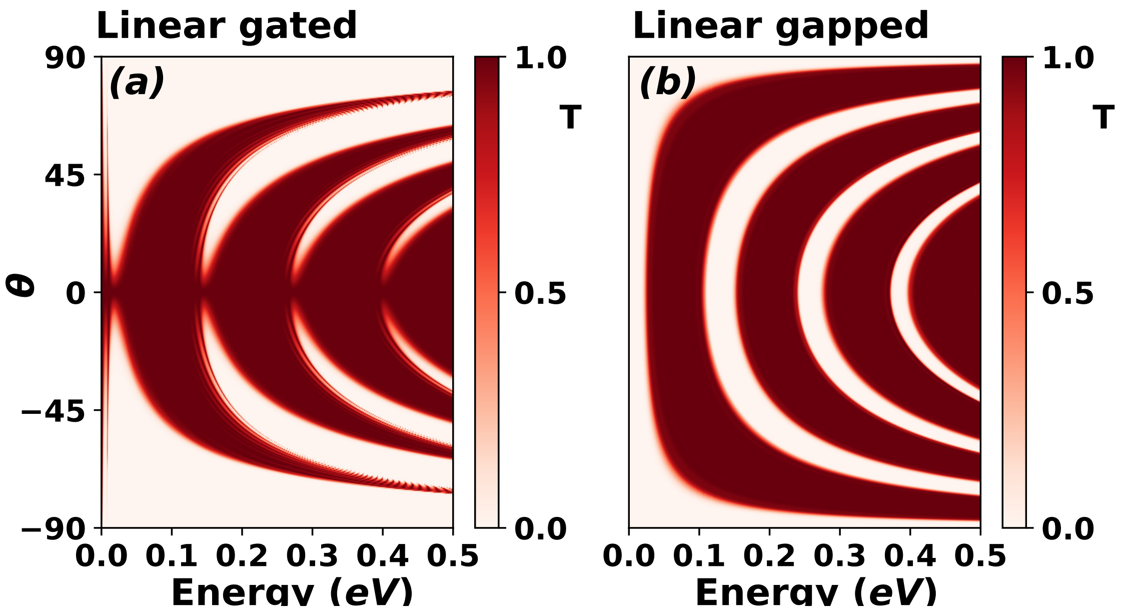

Fig. S28: The same as in Fig. S27, but for Linear GSLs.

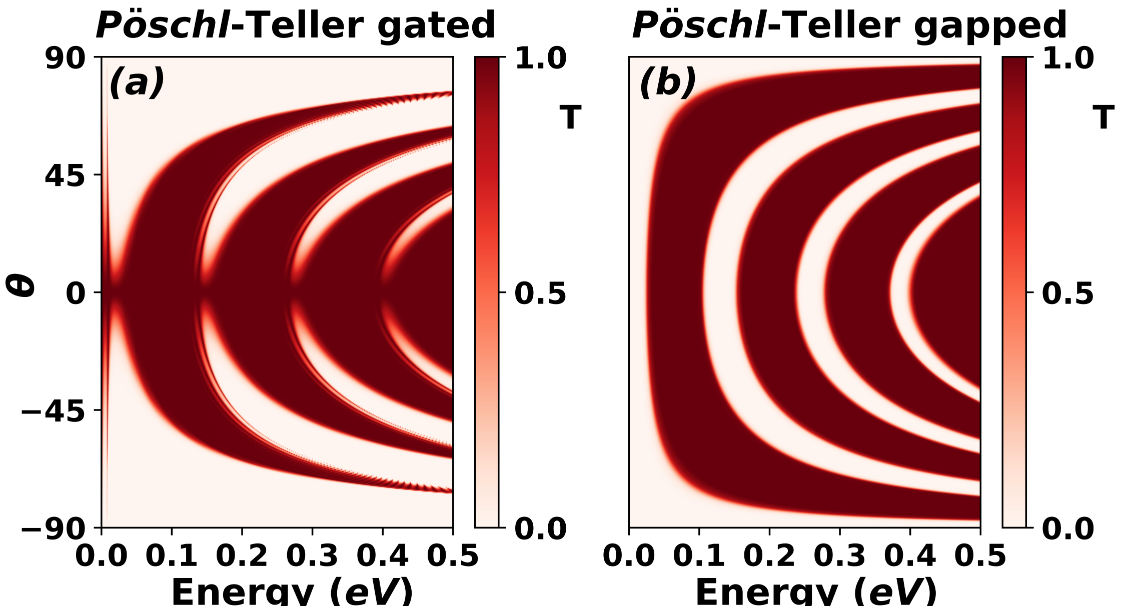

Fig. S29: The same as in Fig. S27 and S28, but for Pöschl-Teller GSLs.
